# Perinatal glucocorticoid sensitivity in the preterm newborn: molecular mechanisms, endogenous determinants, and clinical implications

**DOI:** 10.3389/fendo.2025.1587891

**Published:** 2025-07-16

**Authors:** Nana A. O. Anti, Ciprian P. Gheorghe, Douglas D. Deming, Olayemi O. Adeoye, Lubo Zhang, Eugenia Mata-Greenwood

**Affiliations:** ^1^ Lawrence D. Longo Center for Perinatal Biology, Department of Basic Sciences, School of Medicine, Loma Linda University, Loma Linda, CA, United States; ^2^ Department of Obstetrics and Gynecology, School of Medicine, Loma Linda University, Loma Linda, CA, United States; ^3^ Division of Neonatology, Department of Pediatrics, School of Medicine, Loma Linda University, Loma Linda, CA, United States; ^4^ Department of Pharmaceutical Sciences, School of Pharmacy, Loma Linda University, Loma Linda, CA, United States

**Keywords:** corticosteroids, glucocorticoid sensitivity, glucocorticoid receptor, intrauterine stress, preterm neonate, neonatal morbidity

## Abstract

Glucocorticoids are steroid hormones that regulate multiple physiological processes throughout the lifespan and play a central role in the adaptive stress response. Their biological effects are mediated by the glucocorticoid receptor, which acts through both genomic and nongenomic mechanisms to regulate transcriptional signatures and intracellular signaling pathways, respectively. These effects are tissue- and context-dependent, allowing the body to adapt to developmental and environmental changes. Glucocorticoid-mediated effects are influenced by both hormone bioavailability and tissue-specific responsiveness. Reduced glucocorticoid sensitivity has been observed in patients with severe disease or a diminished response to synthetic glucocorticoid therapies. During the perinatal period, the endogenous glucocorticoid cortisol exerts unique developmental effects on the late-gestation fetus that are essential for extrauterine life. Antenatal glucocorticoid therapy has demonstrated beneficial effects in preventing prematurity-related diseases, while postnatal glucocorticoid treatment reduces inflammation and improves oxygenation in bronchopulmonary dysplasia. However, these therapies exhibit variable responses, both in terms of their beneficial and adverse effects. Furthermore, preterm newborns are exposed to adverse intrauterine environments, including placental insufficiency and infection, which—when combined with immaturity—result in dysregulated perinatal glucocorticoid homeostasis. Intrauterine stressors can therefore alter fetal glucocorticoid sensitivity, partially explaining the variability in clinical outcomes observed among preterm newborns. These adverse conditions may also interact with genetic and physiological factors, such as gestational age and fetal sex, further amplifying glucocorticoid homeostasis dysregulation. In this review, we explore the clinical and basic science evidence on the endogenous determinants of perinatal glucocorticoid sensitivity, with an emphasis on their clinical implications for disease risk and the efficacy of glucocorticoid therapy in the preterm newborn.

## 1 Introduction

Glucocorticoids are essential hormones for vertebrate life that act via the glucocorticoid receptor (GR), a nearly ubiquitously expressed molecule with diverse functions throughout the body (1, 2). Their principal biological effect is the maintenance of glucose homeostasis, in coordination with other metabolic hormones (2). Additionally, they participate in the adaptive stress system in a stressor-dependent manner (2, 3). During pregnancy, glucocorticoids rise at three discrete windows to regulate embryo implantation, adrenal gland development, and fetal organ development necessary for extrauterine life (4, 5). Regarding the latter, synthetic antenatal corticosteroid (ACS) therapy was developed and has proven to be the most effective intervention to prevent preterm-related morbidity (6). A single course of ACS, typically consisting of two 12 mg intramuscular doses of betamethasone administered 24 h apart between 24 and 34 weeks of gestation, is currently accepted by most governing bodies (7–10). In addition, postnatal corticosteroids (PCS), usually low-dose systemic dexamethasone administered after 7 days of life in high-risk preterm newborns, are effective in treating bronchopulmonary dysplasia (BPD) (11, 12). Although perinatal glucocorticoid therapy effectively mitigates complications associated with preterm birth, significant variability in treatment response persists. Endogenous factors, including pregnancy complications such as intrauterine growth restriction (IUGR), maternal diabetes, and chorioamnionitis, are believed to influence ACS efficacy (13, 14). In addition, exogenous determinants—such as type of steroid, timing, and dosing regimen–affect responses to both ACS (5, 15, 16) and PCS (17, 18). Growing awareness has highlighted the clinical significance of these endogenous and exogenous factors in modulating perinatal glucocorticoid sensitivity and influencing neonatal outcomes (19). To this end, we undertook a review of the literature on endogenous factors that influence fetal/neonatal glucocorticoid sensitivity and their impact on short-term outcomes in preterm newborns. This narrative review first outlines key concepts in glucocorticoid physiology and interindividual variability in sensitivity across the general population. It then discusses the primary biological and therapeutic effects of perinatal glucocorticoids before analyzing existing literature on endogenous factors associated with glucocorticoid sensitivity variability in preterm newborns. Our goal is to highlight critical knowledge gaps and encourage future research in this important research field.

## 2 Glucocorticoid physiology

The synthesis and secretion of endogenous glucocorticoids are under the control of the hypothalamic-pituitary-adrenal (HPA) axis, which in turn is regulated by the diurnal circadian rhythm (1, 2). This neuroendocrine axis begins with the hypothalamic secretion of corticotropin-releasing hormone (CRH) and vasopressin that act synergistically to increase the release of adrenocorticotropic hormone (ACTH) from the anterior pituitary gland into the circulation. ACTH subsequently stimulates the adrenal cortex to secrete glucocorticoids (1, 2). Cortisol is the primary endogenous glucocorticoid in humans that classically regulates metabolism. It has been proposed that through its role in energy mobilization, glucocorticoids acquired auxiliary effects in other organ systems involved in the stress response, including the immune, central nervous, and cardiovascular systems (2). Cortisol circulates bound to the corticosteroid binding globulin (CBG), and only ~ 5% unbound cortisol is able to cross the plasma membrane and bind to GR (1, 2). Notably, cortisol also binds to the mineralocorticoid receptor (MR) with a similar affinity to that of the GR (1, 2). However, physiological levels of cortisol do not induce mineralocorticoid effects because 11β-hydroxysteroid dehydrogenase type 2 (11β-HSD2) inactivates cortisol into cortisone in tissues with high MR expression (1). In contrast, organs with high sensitivity to glucocorticoids, such as the liver, adipose tissue, and skeletal muscle, highly express 11β-HSD1, which catalyzes the reverse reaction of 11β-HSD2, thereby increasing cortisol bioavailability (1). Altogether, the regulation of the HPA axis, plasma CBG levels, and tissue expression of 11β-HSD enzymes ultimately determine cortisol bioavailability, which, in conjunction with tissue-specific GR expression and function, dictates the specific biological responses. Synthetic glucocorticoids like dexamethasone and betamethasone were developed to specifically activate GR and thus have little mineralocorticoid activity (2). These synthetic GR agonists, which are resistant to 11β-HSD inactivation, are widely used in pediatric and adult patients as anti-inflammatory agents, as well as in preterm fetuses/newborns to prevent prematurity-related morbidity and mortality.

### 2.1 Glucocorticoid receptor physiology: structure

**Figure 1:**
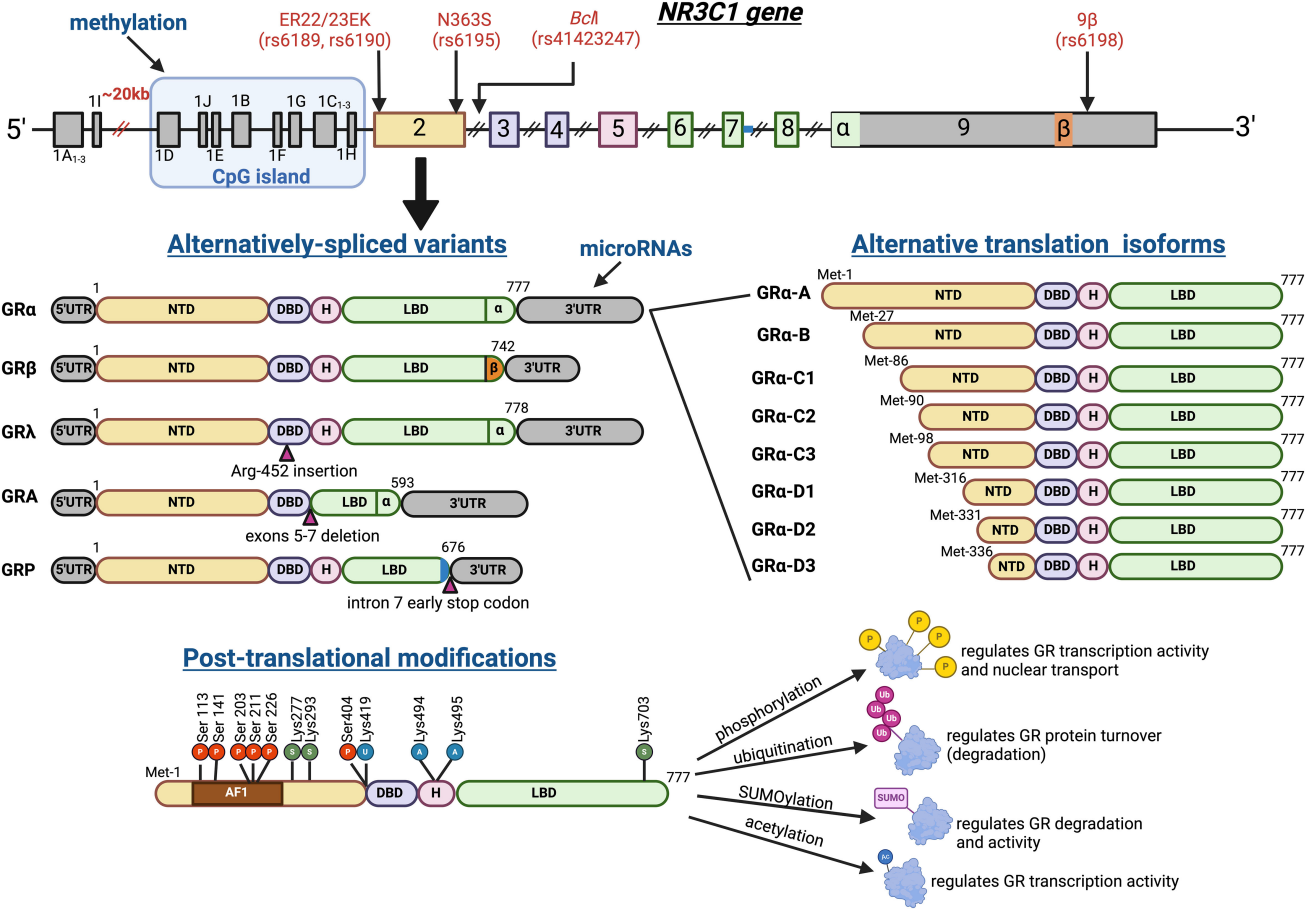
Structure and regulation of the human GR. The *NR3C1* gene, which encodes the human GR, consists of nine alternatively spliced exons and proximal and distal promoter regions. Multiple genetic variants have been reported; shown are four of the most commonly studied polymorphisms. The proximal promoter is embedded in a CpG island known to be regulated by methylation. The proximal and distal promoters contain nine alternative, untranslatable exon 1 variants. Differential promoter usage leads to 5′UTR isoform diversity. In addition, alternative splicing results in five GR transcriptional variants that lead to different protein isoforms: GRα, GRβ, GRγ, GRA, and GRP. The main isoform, GRα, mediates the majority of glucocorticoid biological effects. GRβ has an alternative exon 9, does not bind to cortisol, and is thought to act as a transcriptional inhibitor of GRα. GRγ has an additional codon that translates into an arginine at position 452 and mediates glucocorticoid effects in mitochondria. GRA and GRP have further exon deletions and exhibit reduced and distinct transcriptional profiles compared with GRα. All mRNA isoforms can be regulated epigenetically by microRNAs. Alternative translation protein isoforms for GRα have also been described, resulting in progressively shorter N-terminal domain-containing isoforms with differential transcriptional activities. Posttranslational modifications, including phosphorylation, acetylation, and SUMOylation, regulate GR nuclear translocation and transcriptional mechanisms of action. Finally, ubiquitination targets GRα for proteasomal degradation, thereby limiting glucocorticoid overshooting effects.

GR is encoded by the ~ 126-kb *NR3C1* gene and is composed of nine exons (**Figure 1**) (1, 20). The primary mRNA transcript consists of the 5′ untranslated region (5′UTR) encoded by exon 1, the N-terminal transactivation domain encoded by exon 2, the DNA-binding domain encoded by exons 3 and 4, the hinge region encoded by exon 5, the ligand-binding domain encoded by exons 6–9, and the 3′ untranslated region (3′UTR). The N-terminal domain contains the activation function 1 domain (AF-1) that recruits co-regulators in a ligand-independent manner, while the ligand-binding domain contains a second transactivation domain (AF-2) that serves as a platform for a different set of ligand-dependent coregulators (20, 21). The DNA-binding domain contains two zinc-binding motifs necessary for dimerization and DNA binding, while the hinge region contains two nuclear localization signals needed for transport through the nuclear pore (20, 21). GR is nearly ubiquitously expressed, yet glucocorticoids exert context- and tissue-dependent biological effects partly due to an array of transcript variants and protein isoforms. Alternative splicing of GR mRNA results in splice variants GRα, GRβ, GRγ, GRA, and GRP. Among these, GRα is the primary isoform that mediates the majority of glucocorticoid actions. GRα includes all nine exons and is the most abundant isoform (1, 20). GRβ is also generated by the primary transcript, thereby containing all 9 exons, but differs from GRα on an alternative splice site at the end of exon 8 (1, 20). Therefore, GRβ, by lacking crucial regions of the ligand-binding domain, was previously assumed to be unable to bind glucocorticoids, translocate into the nucleus, or directly regulate gene expression. However, recent evidence demonstrates that GRβ binds to the glucocorticoid antagonist RU486, localizes in both the nucleus and cytoplasm, and modulates gene expression (22, 23). Currently, GRβ is considered a dominant negative inhibitor of GRα by forming inactive heterodimers (1, 22). Remarkably, GRβ abundance is approximately 1,000-fold less than that of GRα, and thus, its contribution to glucocorticoid-mediated biological effects continues to be under debate (20). The GRγ isoform contains a trinucleotide insertion in the boundary of exons 3 and 4, which introduces a single arginine residue at position 452 in between the zinc fingers of the DNA-binding domain. GRγ constitutes ~ 5%–10% of total GR transcripts, has shown impaired transactivation activity, and has been proposed to regulate mitochondrial homeostasis (24, 25). GRA lacks exons 5–7, while GRP is missing exons 8 and 9, resulting in both isoforms lacking important regions of the ligand-binding domain, which limits their function (1, 20). GRA is the most understudied isoform, with only one report of its expression in a myeloma cell line (26). GR-P is upregulated in certain cancers and glucocorticoid-resistant cell lines, suggesting a role of this isoform in decreased glucocorticoid sensitivity (20, 27).

In addition to the splice variants, GR has eight alternative translation initiation sites in exon 2, arising from leaky ribosomal shunting of the mRNA (20). These GR protein isoforms have been observed only in GRα; all of them bind glucocorticoids and regulate gene transcription (20). They include GRα-A, GRα-B, GRα-C1, GRα-C2, GRα-C3, GRα-D1, GRα-D2, and GRα-D3, each with progressively shorter N-terminal domains (**Figure 1**). Interestingly, the overexpression of the individual protein isoforms *in vitro* results in distinct transcriptional regulatory profiles, with only about 10% overlap (28, 29). GRα-A is the canonical full-length GR protein isoform with 777 amino acids and is thought to mediate most of the glucocorticoid functions. GRα-B, with 751 amino acids, has demonstrated increased gene transactivation capacity compared to GRα-A (30). However, GRα-B regulates fewer genes than the full-length isoform, GRα-A, mainly because of its reduced transrepressive efficacy (28). GRα-C1–3 isoforms have 692, 688, and 680 amino acids, respectively. GRα-C1 and -C2 have similar dexamethasone-induced transactivation activity as GRα-A (29). GRα-C3 has the highest *in vitro* transactivation activity of all translational isoforms, which is thought to be mediated by the absence of amino acids 1–97, leading to enhanced recruitment of co-activators to the AF-1 domain (31). GRα-D1–3 isoforms are 462, 447, and 441 amino acids, respectively, and are predominantly localized in the nucleus in both the liganded and unliganded forms, likely due to the absence of amino acids 98–335 that results in conformational changes that expose the nuclear localization signals (32). GRα-D isoforms have the lowest *in vitro* transcriptional regulatory activity. In particular, they demonstrate reduced NF-κB interaction and decreased transrepression of proinflammatory and apoptotic genes compared to other isoforms (29, 32). These GR protein isoforms are regulated throughout development. For example, in the human prefrontal cortex, GRα-D expression is highest in neonates, and it decreases across the lifespan, whereas GRα-A peaks in adolescence (33). In dendritic cells, the more abundant GRα-D isoforms are replaced with GRα-A during maturation, leading to a more glucocorticoid-sensitive phenotype in the mature compared to immature cells (34). Although these studies suggest that expression patterns of GR isoforms contribute to unique cellular responses to glucocorticoids, future *in vivo* studies of GR mRNA/protein isoform overexpression are required to confirm GR isoform-specific functions. Furthermore, research on the mechanisms of GR isoform regulation is needed to advance our understanding of gene pathways involved in disease and glucocorticoid therapy response.

### 2.2 Glucocorticoid receptor physiology: regulation

The expression and function of GR are tightly regulated at the epigenetic, transcriptional, posttranscriptional, and posttranslational levels (**Figure 1**) (21, 35). The *NR3C1* gene has nine alternative untranslated exon 1, each regulated by its own proximal promoter (36) that results in differential tissue expression (37, 38). The alternative first exons are located either in a distal promoter region (1A and 1I), located ~ 30 kb upstream of the translational start site, or a proximal promoter region (1B, 1C, 1D, 1E, 1F, 1H, and 1J), located ~ 0.4–5 kb upstream of the translational start site (35). Furthermore, the proximal promoters are embedded in a CpG island and thereby regulated by methylation and chromatin remodeling, further contributing to differential GR expression regulation (35). Posttranscriptional regulation of GR occurs partly via its 3′UTR region, which is enriched with functional adenylate uridylate-rich elements that recruit RNA-binding proteins, leading to mRNA degradation (35). In addition, various miRNAs such as miR-18, miR-124, and miR-142-3p have also been shown to target the GR 3′UTR for degradation, thereby decreasing glucocorticoid sensitivity (39, 40).

GR protein also undergoes posttranslational modifications that regulate protein stability and function, including ubiquitination, phosphorylation, SUMOylation, and acetylation (**Figure 1**). GR protein turnover is greatly stimulated in response to glucocorticoid binding through ubiquitination and subsequent proteasomal degradation (21). Liganded GR becomes ubiquitinated on the Lys419 residue by E3-ubiquitin ligases, which recruit the proteasome (21). Phosphorylation, a highly researched posttranslational modification, has been identified at Ser113, Ser134, Ser141, Ser203, Ser211, Ser226, and Ser404. GR phosphorylation occurs mostly at residues located in the N-terminal domain that contains the AF-1 domain. Therefore, phosphorylation can affect GR-ligand and DNA-binding activity, thus serving as another mechanism of fine-tuning tissue glucocorticoid response (21). For instance, Ser211 and Ser203 phosphorylation enhance GR transcriptional activity and are required for the full GR-mediated effects, whereas Ser226 and Ser404 phosphorylation promote GR nuclear export and thus limit GR genomic effects (41). Acetylation also regulates GR protein function: ligand binding induces cytosolic-GR acetylation at Lys494 and Lys495 residues, needed for nuclear translocation (42, 43). This is followed by nuclear-GR deacetylation by histone deacetylase 2, a required event for NF-κB transcriptional repression (42, 43). Finally, GR SUMOylation at Lys277, Lys293, and Lys703 has been shown to decrease protein stability, DNA binding ability, and transcriptional activity, leading to decreased GR function (21).

### 2.3 Glucocorticoid receptor physiology: molecular mechanisms of action

#### 2.3.1 Genomic mechanisms of action

**Figure 2:**
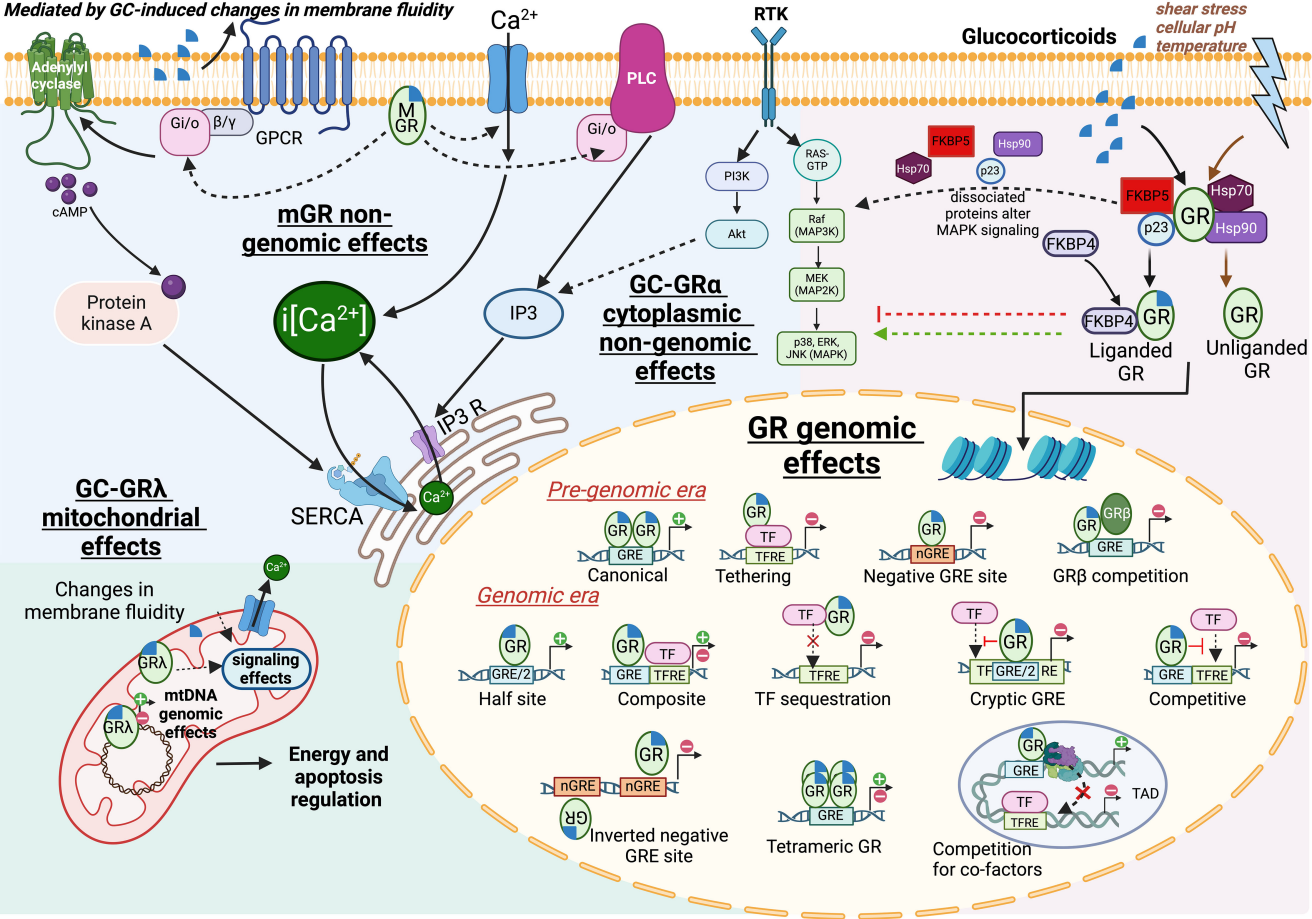
Glucocorticoid molecular mechanisms of action. Cytosolic GR activation by ligand binding leads to the release of chaperones (HSP90, HSP70) and a switch from FKBP5 to FKBP4 that aids in GR nuclear translocation. Other signals, such as a change in cellular pH, can also activate GR in a ligand-independent manner. GR transcription was thought to be regulated by two main mechanisms: GRα homodimer binding to a palindromic glucocorticoid response element (GRE) to induce transactivation, and GRα monomer interaction with other transcription factors leading to transrepression, a process known as tethering. Different genomic mechanisms were later discovered, such as cryptic GRE half-sites, inverted negative GRE sites, and competitive mechanisms in topologically associated domains (TAD). Nongenomic effects of glucocorticoids are thought to be mediated by either an uncharacterized membrane GR (mGR), cytosolic GRα, or mitochondrial GRγ. First, mGR can signal through GTPases, thereby activating or inhibiting key signaling factors like adenylyl cyclase and calcium channels. Second, glucocorticoids can induce fluidity changes in the plasma membrane in a GR-independent manner, which alter the function of transmembrane proteins. Third, cytosolic GRα can interact with other signaling pathways, such as MAPK, either directly or indirectly through dissociated chaperone complex proteins. Lastly, GRγ can regulate mitochondrial functions like ATP synthesis and apoptosis through transcriptional and nongenomic mechanisms. Altogether, glucocorticoids exhibit multiple and complex mechanisms of action that underlie their diverse stimulus- and cell-type-specific biological effects.

GR is localized in the nucleus, cytosol, mitochondria, and cell membrane (**Figure 2**). In the cytosol, GR is mainly found in its inactive form, complexed with chaperones such as HSP90, HSP70, and FKBP5 (1, 20). Upon ligand binding, GR dissociates from these cytosolic chaperones and translocates into the nucleus, where it exerts its genomic effects. Ligand-activated GR can regulate ~ 1%–20% of the transcriptome in an environment- and cell type-specific manner by using multiple mechanisms (1, 20). The classical mechanism consists of liganded GR homodimerization and binding to conserved palindromic DNA sequences known as glucocorticoid response elements (GRE), leading to gene transactivation. Glucocorticoids can also inhibit gene expression through various mechanisms, a research topic of significant interest due to its relevance to the anti-inflammatory effects of synthetic glucocorticoids. In the pregenomic era, it was thought that transrepression by GR did not require DNA binding but was mediated by monomeric GR interactions with other transcription factors, a mechanism known as tethering (44). This central mechanism became the core dogma of the anti-inflammatory effects mediated by glucocorticoids, particularly regarding their interactions with the proinflammatory factor NF-κB. However, in the past decade, GR ChIP-seq and GR transgenic mice studies have uncovered novel breakthrough mechanisms that have enabled a more comprehensive understanding of GR-mediated transrepression apart from tethering. For instance, it is now known that GR dimerization and DNA binding are both needed for full transrepression of proinflammatory genes (45, 46). Furthermore, novel GR genomic mechanisms, including cryptic site monomeric binding at proinflammatory transcription factor sites (47, 48), tetrameric GR binding to GREs (49), and inverted GR binding to nGRE sites (46), have also been discovered (**Figure 2**). Other studies have suggested that rapid (< 1 h) glucocorticoid transrepressive effects are mediated by GR–GRE interactions that sequester cofactors from other transcription complexes, leading to reduced activity of proinflammatory enhancer subsets within shared topologically associated domains (50). This mode of action is highly dependent on chromatin remodeling, which brings different subsets of GRE-containing promoters into close proximity with distant proinflammatory DNA elements. These novel mechanisms can explain why glucocorticoids only inhibit a fraction of NF-κB-responsive genes, why these transrepressive actions require GR–DNA binding, and why they induce different transcriptomic profiles according to cell type and environment. Finally, GR biological effects can also occur in a ligand-independent manner, as demonstrated by studies that showed GR activation in response to physical stressors such as chemicals, cellular pH changes, and temperature fluctuations (51).

#### 2.3.2 Nongenomic mechanisms of action

Glucocorticoids also have rapid onset (seconds to minutes) and short-term (< 90 min) nongenomic effects that are independent of transcription (20) (**Figure 2**). These nongenomic mechanisms mediate glucocorticoid effects in mitochondrial homeostasis, apoptosis, intracellular calcium homeostasis, nitric oxide signaling, and skeletal/smooth muscle function (52). Cytosolic or membrane GR activation can induce rapid changes in different intracellular signaling pathways (52). It has also been proposed that, during receptor activation, dissociation of GR from the chaperones HSP90, HSP70, and FKBP5 may allow the liganded GR to directly interact with MAPK signaling proteins (53). Alternatively, an uncharacterized membrane GR can signal acutely through GTPases to other membrane-associated proteins, such as calcium ion channels, leading to changes in intracellular Ca^2+^ concentrations and other secondary messengers (52). Finally, GRλ has been shown to localize in the mitochondria, where it regulates cellular energy metabolism and apoptotic pathways via both genomic and non-genomic effects (25, 52). In this respect, previous studies have suggested that mitochondrial GR can bind to GRE-like DNA regulatory elements in the mitochondrial DNA and upregulate genes responsible for mitochondrial biogenesis and energy metabolism (52, 54).

## 3 Overview of endogenous determinants of glucocorticoid sensitivity

In healthy populations, longitudinal and cross-sectional studies have shown significant interindividual differences in basal and stressor-specific glucocorticoid sensitivity, typically assessed by cortisol levels and peripheral blood mononuclear cells (PBMC) *in vitro* response to dexamethasone (2, 55). These observational approaches have suggested that glucocorticoid sensitivity variability among healthy individuals is greatly determined by both genetic and early-life stressors (55). In addition, sexual dimorphism in glucocorticoid sensitivity has been conclusively demonstrated in adults and thought to be partially mediated by gonadal hormone regulation of the HPA axis and tissue GR expression and function (56, 57). For instance, premenopausal women in the luteal phase show similar basal free cortisol levels as age-matched men but higher total cortisol and CBG levels (58, 59). Furthermore, some studies have shown that certain stressors elicit a stronger *in vivo* HPA axis response and *in vitro* PBMC sensitivity to dexamethasone-induced repression of pro-inflammatory gene expression in men compared to women (58). Decreased PBMC sensitivity to dexamethasone in women compared to men can be partly explained by lower GR protein expression in T lymphocytes and neutrophils (60). Currently, sexual dimorphism in glucocorticoid sensitivity is thought to partly explain the increased risk of autoimmune diseases in women and the heightened susceptibility to metabolic and infectious diseases in men. Aging also influences glucocorticoid sensitivity: elderly populations exhibit reduced glucocorticoid-mediated inhibition of the HPA axis, higher basal cortisol levels, and lower *in vitro* PBMC sensitivity to dexamethasone compared to younger individuals. However, the underlying molecular mechanisms remain unclear (61). Lastly, and of clinical significance, interindividual variability of glucocorticoid sensitivity has been associated with disease severity and differential response to synthetic glucocorticoid treatment (55, 62). This has prompted extensive research across medical fields aimed at elucidating the mechanisms underlying dysregulated glucocorticoid sensitivity among patients, as discussed below.

### 3.1 Genetic determinants of glucocorticoid sensitivity

Population studies have identified four common genetic polymorphisms in the *NR3C1* gene (**Figure 1**) that are associated with glucocorticoid sensitivity. The *Bcl*I variant (rs41423247) and the nonsynonymous N363S variant (rs6195) are associated with increased clinical glucocorticoid sensitivity, as evidenced by insulin resistance and central obesity (55, 63). Although the molecular mechanisms of increased glucocorticoid sensitivity remain unidentified, there is evidence of increased glucocorticoid sensitivity in cells overexpressing the N363S variant (64). In contrast, the ER22/23EK (rs6189–rs6190) and 9β (rs6198) variants are associated with decreased glucocorticoid sensitivity, as evidenced by improved metabolic parameters such as insulin sensitivity and muscle mass (55, 65). A potential mechanism for decreased glucocorticoid sensitivity in ER22/23EK variant carriers is the predominant expression of the GRα-A isoform, compared to the more sensitive GRα-C3 protein isoform, as demonstrated by *in vitro* overexpression assays (66). Conversely, carriers of the 9β variant have shown decreased GR transrepressive efficacy *in vitro* (67), in association with increased GRβ stability. This polymorphism has resurfaced as a clinically relevant biomarker of decreased response to synthetic glucocorticoid therapy in several diseases, such as childhood lymphoblastic leukemia (68) and coronavirus disease 2019 (COVID-19) (69). Overall, *NR3C1* gene variants have modest contributions to metabolic phenotypes and glucocorticoid therapy efficacy. Current ongoing research approaches, such as the use of genome-wide association studies with RNA sequencing, are likely to unveil the complex gene–gene and gene–environment interactions and provide novel genetic biomarkers of glucocorticoid sensitivity.

### 3.2 GR expression as a determinant of glucocorticoid sensitivity

Perhaps the most widely researched and accepted endogenous determinant of tissue glucocorticoid sensitivity is GR levels, particularly in correlation with clinical responses to synthetic glucocorticoid therapy (55, 62). The main approach consists of examining GR expression before (baseline) and after glucocorticoid treatment in glucocorticoid-sensitive and glucocorticoid-resistant patients, classified according to symptom resolution. Lower baseline GR protein levels were observed in glucocorticoid-resistant compared to glucocorticoid-sensitive patients in pediatric asthma, adult immune thrombocytopenia, adult rheumatoid arthritis, adult interstitial lung disease, and adult systemic lupus erythematosus (55, 62, 70). Other studies reported significant differences between glucocorticoid-sensitive and -resistant patients in baseline GR mRNA isoforms; for instance, higher baseline GRβ in severe adult asthma (71) and higher GRλ in pediatric leukemia non-responders compared to responders (72). Therefore, GR expression is an important determinant of clinical outcomes and therapy response in such diseases. Conversely, other studies found similar baseline GR levels in both glucocorticoid-sensitive and glucocorticoid-resistant patients of adult asthma (73), chronic obstructive pulmonary disease (74), idiopathic nephrotic syndrome (75), and Vogt–Koyangi–Haradi syndrome (76), suggesting alternative GR-expression-independent mechanisms of glucocorticoid resistance. Presently, the mechanisms that lead to decreased GR expression in glucocorticoid-resistant patients remain mostly undefined, although unchecked inflammation has been proposed as a principal mechanism (55, 63). Altogether, these studies highlight the complex interaction of disease-specific factors in regulating GR expression and the limitations of studying GR number and affinity as a sole mechanism of glucocorticoid sensitivity. Moreover, the interventions to normalize GR expression in patients at risk of glucocorticoid resistance warrant future research.

### 3.3 GR function as a determinant of glucocorticoid sensitivity

PBMCs, or other available sources such as tissue biopsies, have been used to examine differential GR function in association with disease and glucocorticoid therapy. Similar to GR expression studies, *in vitro* cell-based approaches also showed correlation with clinical outcomes. Indeed, functional baseline *in vitro* dexamethasone resistance correlated with glucocorticoid resistance in patients with pediatric/adult asthma (70, 71), rheumatoid arthritis (55), systemic lupus erythematosus (55), chronic obstructive pulmonary disease (74), pediatric leukemia (77), alcoholic hepatitis (78), and inflammatory bowel disease (79). Importantly, novel mechanisms of decreased GR function, such as p38 MAPK activation, IL17A activation, and vitamin D deficiency, were uncovered with the aid of these cell-based assays (73, 80, 81). These discoveries led to clinical trials that improved clinical outcomes and glucocorticoid response in vitamin D-supplemented asthmatic patients (82). In addition, cell-based functional assays have been useful to study determinants and mechanisms of disease severity. For instance, *in vitro* dexamethasone resistance was observed in pediatric leukemia patients younger than 1.5 years in association with worse survivorship (77). Other studies revealed a decreased *in vitro* PBMC response to dexamethasone in correlation with the severity of major depressive syndrome (83) and sepsis (84), while no relationship was observed with coronary artery disease (85, 86), and equivocal results were reported with posttraumatic stress syndrome (87).

### 3.4 Cellular environment as a determinant of glucocorticoid sensitivity

The past decades have witnessed a surge in state-of-the-art omics strategies for the uncovering of tissue- and environment-specific molecular mechanisms of glucocorticoid sensitivity in patients requiring glucocorticoid therapy (88, 89). For example, tissue-dependent RNA-seq uncovered decreased glucocorticoid sensitivity in bronchoalveolar lavage immune cells compared to PBMCs in patients with asthma and COVID-19 (90), suggesting pulmonary-specific mechanisms of glucocorticoid resistance. Omic approaches have also aided in untangling complex disease-specific mechanisms of action. For instance, proteomics and metabolomics approaches have identified inhibition of neutrophil and platelet degranulation as a key mechanism of dexamethasone in COVID-19 patients (91), while multigenomic approaches have uncovered polymorphisms in glucocorticoid target genes associated with variability in gene expression regulation in pediatric asthma patients (92). Furthermore, integrated genomic (mRNA, polymorphisms) and epigenomic (miRNA, methylation) strategies have identified novel candidate genes for tailored dosing regimens, novel drug design, and synergistic drug combination therapies (93, 94). For example, an integrated multiomics study in acute lymphoblastic leukemia identified a novel regulatory gene that recapitulated drug resistance *in vitro* and led to a novel synergistic drug combination likely to improve clinical outcomes (94). These approaches have advanced the field of glucocorticoid sensitivity beyond GR-dependent mechanisms to include the role of the tissue-specific environment. Future integrated omics studies are expected to uncover the complex interplay between epigenetic chromatin remodeling and genetic variants in regulating tissue- and context-specific glucocorticoid sensitivity.

## 4 Perinatal glucocorticoid physiological and therapeutic effects

**Figure 3:**
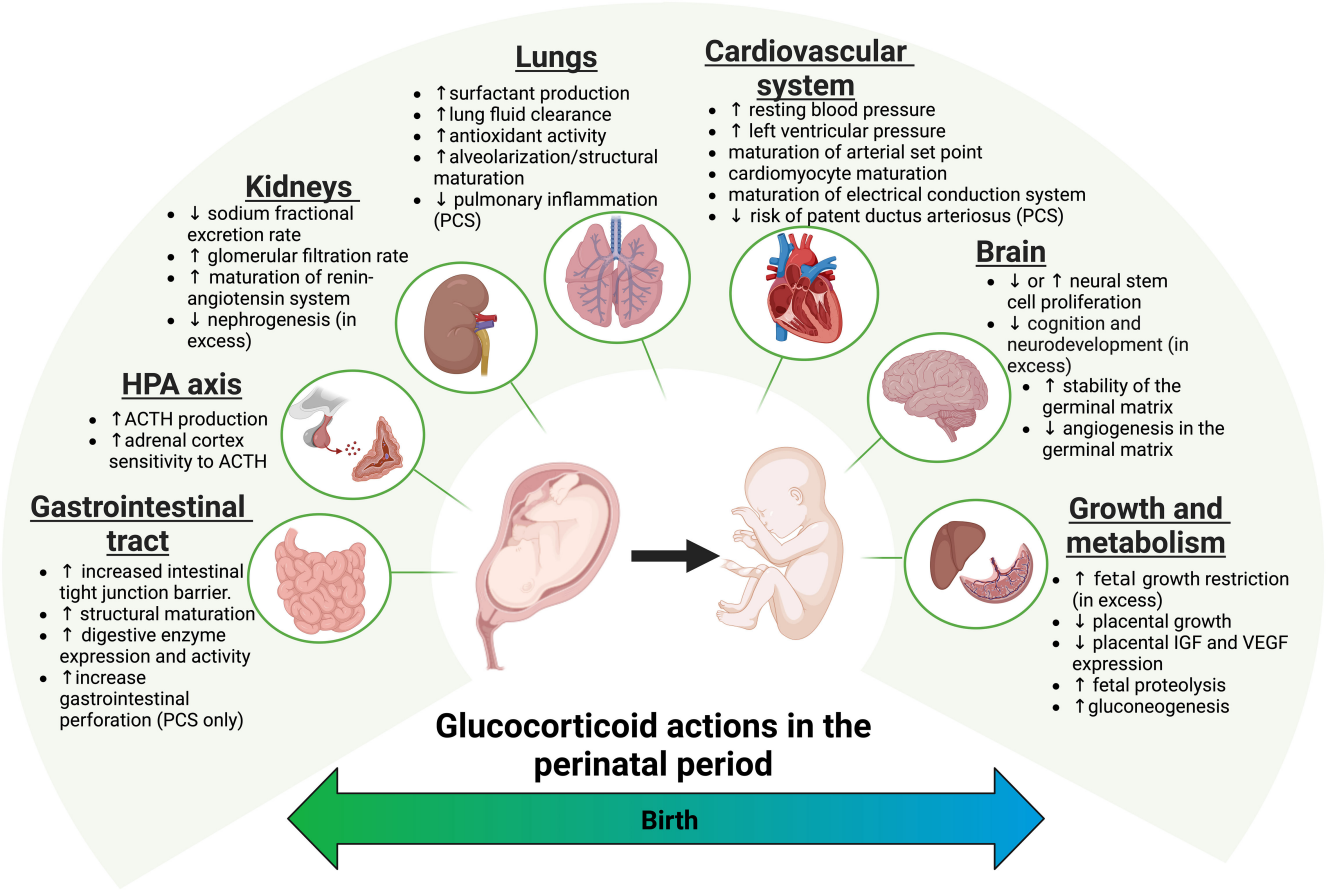
Physiological and therapeutic effects of glucocorticoids in the perinatal period. The physiological surge in glucocorticoids toward the end of pregnancy promotes a sequence of maturational events that prepares the fetus for extrauterine life. Animal studies revealed a vital and primary role of glucocorticoids and GR in lung maturation, which led to the therapeutic use of antenatal corticosteroids in pregnancies at risk of preterm birth. Additionally, glucocorticoids contribute to the maturation of other fetal organs, including the brain, HPA axis, metabolic organs, cardiovascular system, gastrointestinal system, kidneys, and immune system. In the preterm newborn, however, the perinatal effects of both endogenous and synthetic glucocorticoids depend on a combination of exogenous factors, such as timing, type of steroid, and dosage, as well as endogenous factors, including genetic makeup, gestational age, fetal sex, and the presence of concomitant perinatal complications.

In most mammalian species studied to date, there is a dramatic surge in maternal and fetal cortisol towards the end of pregnancy, preparing the fetus for extrauterine life (95). This surge occurs through a complex interplay between the placenta and both maternal and fetal HPA axes (95). By the end of the second trimester, the placenta increases the secretion of CRH into the fetal circulation, where it stimulates the fetal HPA axis, resulting in increased cortisol (99). Concurrently, the placental CRH also stimulates maternal ACTH release, elevating maternal cortisol levels, which can saturate the placental 11β-HSD2 and thus cross unaltered into the fetal circulation (95). Paradoxically, cortisol does not inhibit placental CRH production as it does with hypothalamic CRH, creating a positive feedback loop that drives cortisol production until the end of pregnancy (95). Elevated fetal cortisol levels act as a molecular switch, regulating fetal growth and development by triggering a wide range of physiological changes in key organs, such as the lung, brain, heart, and kidney, helping the fetus adapt effectively after birth (**Figure 3**) (96). While not fully described in the human fetus, the ontogeny of the cortisol receptors (GR and MR) and bioavailability enzymes (11β-HSD1/2) in mice demonstrates physiological coordination in the expression of these genes with cortisol developmental effects (97, 98). Importantly, placental 11β-HSD2 plays an undisputed and critical role as a physiological barrier, protecting the fetus from excessive exposure to high levels of maternal cortisol before the late-pregnancy surge. This enzyme is widely expressed in most embryonic tissues, as well as in the placenta and extraembryonic membranes throughout gestation (97). In contrast, GR expression is low in earlier embryonic developmental stages, with mid-gestation increases in key organs, primarily in the lung, thymus, and pituitary gland, and secondarily in the liver, intestine, muscle, heart, and placenta (98).

The physiological and therapeutic effects of perinatal glucocorticoids have been mostly elucidated with the aid of *in vivo* models of glucocorticoid deficiency, such as adrenalectomy, GR gene deletion or mutation, and synthetic glucocorticoid exogenous administration. In this respect, it is worth mentioning some limitations of these methods. For instance, GR gene manipulation strategies do not provide information on tissue-specific regulation of GR expression and function, and adrenalectomy is limited by timing and subject choice (fetal versus maternal). Exogenous glucocorticoid administration at earlier gestational ages (untimely) or at supraphysiological levels (excess) approaches are limited by the type of steroid, dosing, and timing. In addition, interspecies differences in gestational length and fetal maturational programs represent important limitations when extrapolating data to humans. This section illustrates the complex cellular and molecular mechanisms underlying glucocorticoid perinatal effects and highlights the dynamic role of gestational age as a primary regulator of these effects.

### 4.1 Perinatal glucocorticoid effects in the lung

Glucocorticoids play a critical role in the development of the pulmonary system in the late-gestation fetus. While investigating the role of cortisol in parturition, Liggins serendipitously discovered that antenatal cortisol infusion increased pulmonary surfactant production and aeration in preterm lambs (99). Antenatal glucocorticoid administration has since been proven to induce fetal lung maturation in all mammals studied, including humans (100–103). Shortly after the first randomized controlled trial (RCT) that demonstrated ACS efficacy in preventing neonatal respiratory distress syndrome (RDS) in humans, numerous research strategies aimed to identify the mechanisms underpinning glucocorticoid effects in fetal lung development. Initial studies demonstrated dexamethasone binding in both nuclear and cytosolic fractions of the fetal lung as early as 12 weeks of gestation, pointing to the capacity of the premature fetus to respond to antenatal glucocorticoid treatment (104, 105). Transgenic mouse models then confirmed an essential role of GR in fetal pulmonary development, with GR-null mice showing severe respiratory distress and atelectasis shortly after birth (106–109). Pulmonary morphology was characterized by alveolar septal hypercellularity and reduced air spaces (106–109), with corresponding upregulation of cell proliferation gene pathways (106). GR^dim^ transgenic mice, which carry a mutation that prevents GR dimerization and GRE-dependent transactivation, survived into adulthood without signs of impaired lung development (110). This pivotal study demonstrated that the classical GR genomic mechanism was not essential for lung development, suggesting that nonclassical GR mechanisms, such as transrepression, are required in glucocorticoid-induced lung maturation. Subsequently, tissue-specific GR knockout models demonstrated that only the mesenchymal GR knockout mice recapitulated the RDS lung phenotype observed in GR-null mice (111–113). Furthermore, acute glucocorticoid treatment, administered 48 h before delivery, was sufficient to induce fetal lung maturation in the preterm lamb, as evidenced by increased pulmonary airspace and lung compliance, without affecting surfactant production (114–116). These studies demonstrated that glucocorticoids stimulate pulmonary maturation primarily by mesenchymal-induced structural remodeling, with a secondary effect on surfactant production. Strikingly, generalized inherited glucocorticoid resistance, a rare genetic disorder caused by GR mutations that reduce GR function, does not lead to neonatal RDS (117), suggesting that the mere presence of GR, rather than its full functionality, is sufficient for perinatal lung maturation. These observations highlight the old concept of GR working as a “permissive” transcription regulator (116), currently restated as a “pioneering” factor (46), in synchrony with other transcriptional and chromatin-remodeling factors to drive context-specific biological effects. The critical role exerted by the cellular environment on GR function may explain the intrasubject variability in ACS response observed in rabbit and sheep models, where ~ 40% of preterm animals fail to show improved lung compliance and oxygenation (99, 101, 118). Such endogenous variability in ACS response may stem from interindividual differences in cell-specific chromatin states and GR-interacting transcriptional coregulator pools. Future studies are needed to elucidate the cell-specific GR-interactome in fetal lung across gestational ages pertinent to ACS therapy.

The molecular mechanisms of glucocorticoids in pulmonary development can be broadly summarized as (1) inducing structural maturation of lung tissue (2), enhancing fluid resorption (3), stimulating surfactant production, and (4) increasing the expression of antioxidant enzymes (119). Firstly, and currently considered the most important glucocorticoid effect in lung development, is the promotion of structural maturation in the lungs as shown by expansion of parenchymal air spaces and lung volume, mostly achieved by thinning of the alveolar wall mesenchyme to allow gas exchange (111–113). This effect occurs through reduced cellular proliferation and increased apoptosis of mesenchymal cells (106). Secondly, glucocorticoids enhance lung fluid resorption by stimulating type I alveolar epithelial cell expression of epithelial sodium channels and Na^+^/K^+^ ATPase (120), which allow the removal of alveolar luminal Na^+^ and water. Thirdly, glucocorticoids stimulate surfactant production by various mechanisms, including the differentiation of type II alveolar epithelial cells (121, 122), the induction of surfactant protein expression (121, 123), and increased phospholipid synthesis enzyme activity (124). Finally, glucocorticoids also protect the neonatal lung from oxidative damage caused by postnatal exposure to higher oxygen levels by increasing the activity and expression of antioxidant enzymes such as glutathione peroxidase, superoxide dismutase, and catalase (125, 126).

In addition to the beneficial effects of ACS in preventing RDS, PCS is also effective in preventing and treating BPD (11, 12), which is characterized by the expansion and activation of pro-inflammatory cells that release injurious mediators that further disrupt alveolarization and remodel the immature lung (127). Consequently, PCS treatment has been shown to downregulate proinflammatory mediators and reduce the number of neutrophils in the tracheobronchial aspirates of BPD preterm neonates, leading to earlier extubation and improved disease resolution (128).

### 4.2 Perinatal glucocorticoid effects on growth

Proper fetal growth is regulated by the balance of hyperplasia, hypertrophy, and differentiation processes. Disruptions of this balance can result in growth restriction or macrosomia, both of which are associated with fetal and neonatal morbidity and programming of adult disease (129, 130). In humans, normal physiological levels of cortisol stimulate fetal organ maturation without affecting growth. Indeed, GR deletion models demonstrate normal fetal growth (107, 108), suggesting that glucocorticoids are not essential for fetal cellular proliferation or hypertrophy. Conversely, prenatal exogenous administration of glucocorticoids results in IUGR in a species-, dose-, and gestational age-dependent manner in all researched mammals (116, 129, 130). Notably, single-dose ACS-induced IUGR is more pronounced in small mammals, such as rats and rabbits, compared to sheep and non-human primates, due to the shorter gestational length of rodents relative to higher mammals (116, 131). Furthermore, repetitive doses of ACS result in a more prominent IUGR than a single-dose ACS, accompanied by further acceleration of lung maturation, in both rabbits and sheep (132, 133). ACS induced symmetrical IUGR in rodents and sheep (133–136) but caused brain-sparing asymmetrical IUGR in non-human primates (137–139). Remarkably, meta-analysis studies reported no effect of ACS on human fetal growth (6), which is hypothetically attributed to the short interval between ACS exposure and birth. Supporting this theory, a longitudinal birth cohort study in term newborns revealed that a single course of ACS administered ~ 4 months before term delivery led to smaller birthweights and percentiles in ACS-treated compared to non-ACS-treated newborns (140). Postnatally, supraphysiological levels of glucocorticoids continue to inhibit growth, resulting in short stature with equivocal effects on fat mass and obesity (141).

Glucocorticoids decrease fetal growth directly by inducing fetal proteolysis and indirectly by decreasing placental weight (129, 130). Glucocorticoid effects on fetal growth are partly mediated by other hormones and growth factors, particularly insulin-like growth factors (IGFs) (129). IGFs are known to stimulate tissue growth, playing a crucial role in fetal development. Deletions or mutations of these genes significantly reduce body weight in both rodent models and humans (142, 143). Cortisol levels are negatively associated with fetal IGF expression, an effect likely caused by GR-mediated transrepression (144, 145). In addition, glucocorticoids reduce placental vascularity by downregulating vascular endothelial growth factor and prolactin (146, 147). Postnatally, glucocorticoids stunt growth from birth through adolescence by suppressing the neuroendocrine growth hormone axis (148).

### 4.3 Perinatal glucocorticoid effects in the brain

GR deletion in the brain does not affect brain morphology or cause fetal or early postnatal death, but it results in postnatal behavioral dysfunction (149–151). Similarly, maternal adrenalectomy does not significantly alter brain morphology but delays the differentiation and migration of neurons and overall functional maturation of brain structures such as the hippocampus (152, 153). The physiological effects of glucocorticoids in the brain are mediated by both the GR and MR (154). While GR is distributed throughout all brain regions starting at mid-gestation, MR expression is confined to the hippocampus and limbic system, particularly during late gestation, and is expressed at higher levels than GR (97, 98, 155). In addition, significant increases in both receptor densities concomitantly with reduced 11β-HSD2 and increased cortisol bioavailability occur in late gestation (97, 98, 155). Consequently, cortisol developmental effects in the near-term fetus are mediated mostly through the MR in the limbic system and through GR in other brain regions such as the choroid plexus.

Untimely or excess antenatal glucocorticoids that escape 11β-HSD2 surveillance induce deleterious effects in the fetal brain, leading to postnatal learning disabilities, attention-deficit disorders, anxiety, and depression (97, 154). These adverse effects have been shown to be mediated exclusively through GR stimulation. For instance, dexamethasone and high-dose corticosterone treatment, but not low-dose corticosterone, reduced cell proliferation in embryonic rat neural stem cells *in vitro* through GR activation with no participation from MR (156). Compelling evidence has established that glucocorticoid overexposure leads to decreased brain weight and inhibition of neurogenesis in the cortex, subventricular zone, and regions of the hippocampus, such as the corpus ammonis, dentate gyrus, and subgranular zone (157–159). Glucocorticoids inhibit neurogenesis primarily by decreasing proliferation and increasing apoptosis of neuronal stem cell progenitors. At the molecular level, glucocorticoids upregulate cell-cycle inhibitors and senescence markers while downregulating cell-cycle proteins such as cyclin D1 (156, 160). Downregulation of transcription factors WNT and sonic hedgehog by glucocorticoids has also been implicated in neurogenesis inhibition (161, 162). Furthermore, excessive glucocorticoids caused by repetitive ACS dosing induced deleterious structural changes, including decreased nerve myelination in the corpus callosum and delayed maturation of astrocyte tight junctions (116, 163). Unexpectedly, glucocorticoids have been shown to promote neurogenesis *in vitro* under certain conditions. For instance, Ninomiya et al. demonstrated that glucocorticoids stimulate the proliferation of neural progenitor cells derived from human fetal lung fibroblasts (164). Likewise, Krontira et al. found that glucocorticoids enhanced the proliferation of specific neuronal progenitor subpopulations within brain organoids derived from human skin fibroblasts and glioblastoma cells (165). They proposed that this model reflects active neurogenesis during cortical expansion in the brain and hypothesized that glucocorticoids can induce beneficial effects on neurogenesis during these earlier gestational stages (22–27 weeks). Notably, a recent meta-analysis study found that ACS treatment in extremely preterm newborns was associated with a significantly lower risk of neurodevelopmental impairments compared to those receiving ACS at later gestational ages (166).

Repeated ACS or high-dose PCS regimens have also been associated with increased risk of cerebral palsy (167, 168), partly by aggravating the vulnerability of the developing brain to hypoxia/reperfusion damage in the immature brain. The mechanisms include hypomyelination resulting from oligodendrocyte maturation inhibition and reactive astrogliosis (169). These events render the immature brain more vulnerable to free radical-, glutamate-, and cytokine-induced injury (170). Furthermore, glucocorticoids have been shown to heighten calcium signaling, disrupt glucose uptake, dysregulate mitochondrial function, and enhance glutamatergic signaling, all of which predispose neurons to metabolic insults such as hypoxia (171).

Lastly, ACS also reduces the risk of intraventricular hemorrhage (IVH) in preterm neonates (6). IVH results from immaturity of the germinal matrix substructures combined with fluctuating cerebral blood flow and coagulation disorders, primarily caused by RDS-induced hypoxemia (171, 172). Apart from the beneficial effects of glucocorticoids in preventing RDS-dependent hypoxemia, they also stabilize the germinal matrix capillary network by increasing extracellular matrix protein expression and enhancing pericyte and astrocyte end-feet coverage of vessels (173). In addition, glucocorticoids inhibit endothelial cell proliferation and downregulate vascular endothelial growth factor and angiopoietin 2, further stabilizing the germinal matrix vasculature (173, 174).

### 4.4 Perinatal glucocorticoid effects in the HPA axis

While neuron-specific GR deletion results in mild HPA axis dysregulation, anxiety, and depression-like symptoms (149–151), GR deficiency in both the brain and pituitary gland results in hypercortisolemia, HPA axis dysregulation, growth restriction, and neonatal death within 1–2 weeks after birth (175), highlighting the vital role of glucocorticoids in HPA axis development and regulation. In the sheep, the near-term fetal cortisol surge has been associated with pro-opiomelanocortin mRNA upregulation and ACTH release (176), although the underlying mechanisms remain unknown. Furthermore, exogenous low-dose dexamethasone treatment at earlier gestational ages—corresponding to 34–36 weeks of gestation in humans—elevated both the basal and stress-induced cortisol setpoints, leading to an enhanced response to hypoxemic stress (177). Similarly, human term newborns exposed to ACS at preterm stages exhibited a stronger HPA axis response to a heel-stick procedure (178). It is proposed that the underlying mechanism involves ACS-induced downregulation of hypothalamic and pituitary GR expression, thereby blunting the rapid negative feedback mechanism mediated by cortisol. This disruption then leads to increased and sustained cortisol secretion. In contrast, the effects of ACS in preterm newborns (< 32 weeks of gestation) appear to be opposite to those observed in late-preterm/term newborns. Indeed, ACS-exposed preterm newborns exhibited a blunted HPA axis response to various stressors applied in early postnatal days (179–181). Furthermore, it has been proposed that a switch between hypoactivity and hyperactivity occurs postnatally in preterm populations (182), which is associated with long-term increased basal cortisol levels and metabolic side effects such as obesity. Currently, the mechanisms by which ACS affects the programming of the preterm newborn’s HPA axis remain obscure, with a major obstacle being the lack of representative viable very preterm subjects in nonhuman species. Lastly, preterm newborns often present relative adrenal insufficiency that compounds the demands of critical illness, often resulting in life-threatening hypoglycemia and hypotension (183, 184). In this respect, hydrocortisone, in supplementary doses, is an effective postnatal treatment in preterm newborns < 28 weeks of gestation presenting adrenal insufficiency compounded with circulatory collapse (183, 184).

### 4.5 Perinatal glucocorticoid effects in the cardiovascular system

Glucocorticoids play important roles in the development and maturation of the fetal cardiovascular system, as GR-null mice have small, underdeveloped hearts with impaired function (185). Some of the mechanisms by which glucocorticoids mediate fetal heart development include the following (1): increased resting blood pressure without sustained bradycardia (2), maturation of the arterial setpoint and heightened sensitivity to stressors like hypoxia (3), cardiomyocyte maturation through hypertrophy and binucleation (4), increased left ventricular pressure, and (5) maturation of the electrical conduction system (186, 187). Early in pregnancy, fetal hypoxia induces transient bradycardia and increased peripheral vascular resistance (188). As the fetus approaches term, the glucocorticoid surge further enhances the response to hypoxia, resulting in a stronger and more sustained bradycardic response, along with increased peripheral vascular resistance (188). These maturational effects enhance oxygen extraction, maintain cardiac output, and redistribute blood flow toward vital organs such as the brain (186). Postnatally, PCS can reduce the risk of patent ductus arteriosus (189). The underlying mechanism involves increased sensitivity of the ductal tissue to oxygen-induced vasoconstriction and decreased sensitivity to the vasodilatory effect of prostaglandin E2 (190, 191). However, while glucocorticoids have beneficial physiological effects, excessive and untimely exposure to cortisol or ACS has been implicated in fetal cardiovascular programming, leading to long-term postnatal adverse effects such as hypertension, increased aortic stiffness, and endothelial dysfunction (192).

### 4.6 Perinatal glucocorticoid effects in other organs

The fetal gastrointestinal system responds to glucocorticoids by increasing the expression of secreted protective barrier proteins in the ileum, such as mucin2 and tight junction proteins (193). These effects are believed to contribute to the protective role of ACS against necrotizing enterocolitis (NEC) in preterm newborns (6). The molecular mechanisms mediating these effects involve the upregulation of surfactant protein D in the ileum, which in turn stimulates the expression of tight junction proteins such as occludin and zonula occludens-1 (193). Additional developmental changes include the increased expression and activity of digestive enzymes, along with structural maturation necessary for increased nutrient absorption (192, 193). Conversely, PCS has been associated with gastrointestinal perforation, a rare but potentially life-threatening condition (11, 194). One potential mechanism involves the downregulation of prostaglandin E2, which disrupts gastrointestinal integrity by decreasing mucus and bicarbonate secretion and reducing blood flow (194).

The fetal liver is also sensitive to the developmental effects of glucocorticoids. Indeed, hepatocyte-GR null mice display dysregulated glucose metabolism and decreased gluconeogenesis, with 50% neonatal mortality occurring within 48 h of birth (195). However, excessive glucocorticoid exposure could be detrimental to fetal liver development. For instance, prolonged exposure to dexamethasone during fetal development inhibits liver cell proliferation, leading to abnormal growth and dysplasia in rats (196).

Renal maturational effects stimulated by glucocorticoids include a decreased sodium fractional excretion rate, an increased glomerular filtration rate, and accelerated development of the renin–angiotensin system (197). These effects prepare the fetus for proper postnatal regulation of blood pressure, volume, and mineral homeostasis. Interestingly, GR deletion in the distal nephron does not impair kidney development or function (198), suggesting that cortisol may stimulate renal maturation partly through MR. In contrast, glucocorticoid excess—particularly early in gestation—is associated with reduced nephrogenesis and altered renin–angiotensin axis function (199, 200), and is hypothesized to contribute to the glucocorticoid programming of adult renal and cardiovascular disease.

In the immune system, glucocorticoids have profound and time-sensitive developmental effects (201). During early to mid-gestation, GR is downregulated in hematopoietic tissues such as the liver and thymus, with a concomitant increase in liver 11β-HSD2 expression (97, 98), thereby reducing cortisol bioavailability and its downstream effects. Exposure to excess glucocorticoids during these gestational ages has been shown to impact the migration, proliferation, and differentiation of hematopoietic cell lineages (201). For instance, betamethasone treatment of human fetal liver cells isolated at 7–12 weeks of gestation significantly decreased proliferation of hematopoietic stem cells in a dose-dependent manner (202). Similarly, dexamethasone treatment of mouse fetal liver explants significantly promoted the differentiation of immature hematopoietic cells into myeloid cells at the expense of lymphoid cells, particularly B lymphocytes (203). This observation may explain the reduced B-cell humoral response to vaccination observed in neonates—both preterm and term—exposed to ACS or excess cortisol due to maternal stress (204, 205). In late gestation, however, glucocorticoids have essential physiological effects on thymus development, as well as on thymocyte selection and differentiation. In this respect, GR levels are elevated in the mouse thymus during mid- to late gestation (98), and thymic epithelial cells are known to locally produce glucocorticoids—underscoring the importance of glucocorticoid-GR signaling in the thymus during this late gestational window (206). Remarkably, both GR deletion and excess glucocorticoid exposure result in reduced thymic volume and thymocyte number, although these two conditions induce different immune phenotypes (207–209). Firstly, GR deletion or reduced glucocorticoid bioavailability promotes apoptosis of double-positive thymocytes, leading to a limited repertoire of T cells that are hyporesponsive to antigens and TCR-mediated apoptosis (210–212). In contrast, excess glucocorticoids deplete both double-negative and double-positive thymocytes, leading to accelerated repopulation and selection of immature precursors that are potentially autoreactive, possibly contributing to increased risk of autoimmune diseases later in life (208). Furthermore, glucocorticoid excess also promotes CD4^+^ T helper 2 cell polarization (201), which may explain the higher rates of atopic diseases in neonates exposed to ACS or maternal stress (213, 214).

## 5 Endogenous determinants of perinatal glucocorticoid sensitivity

**Figure 4:**
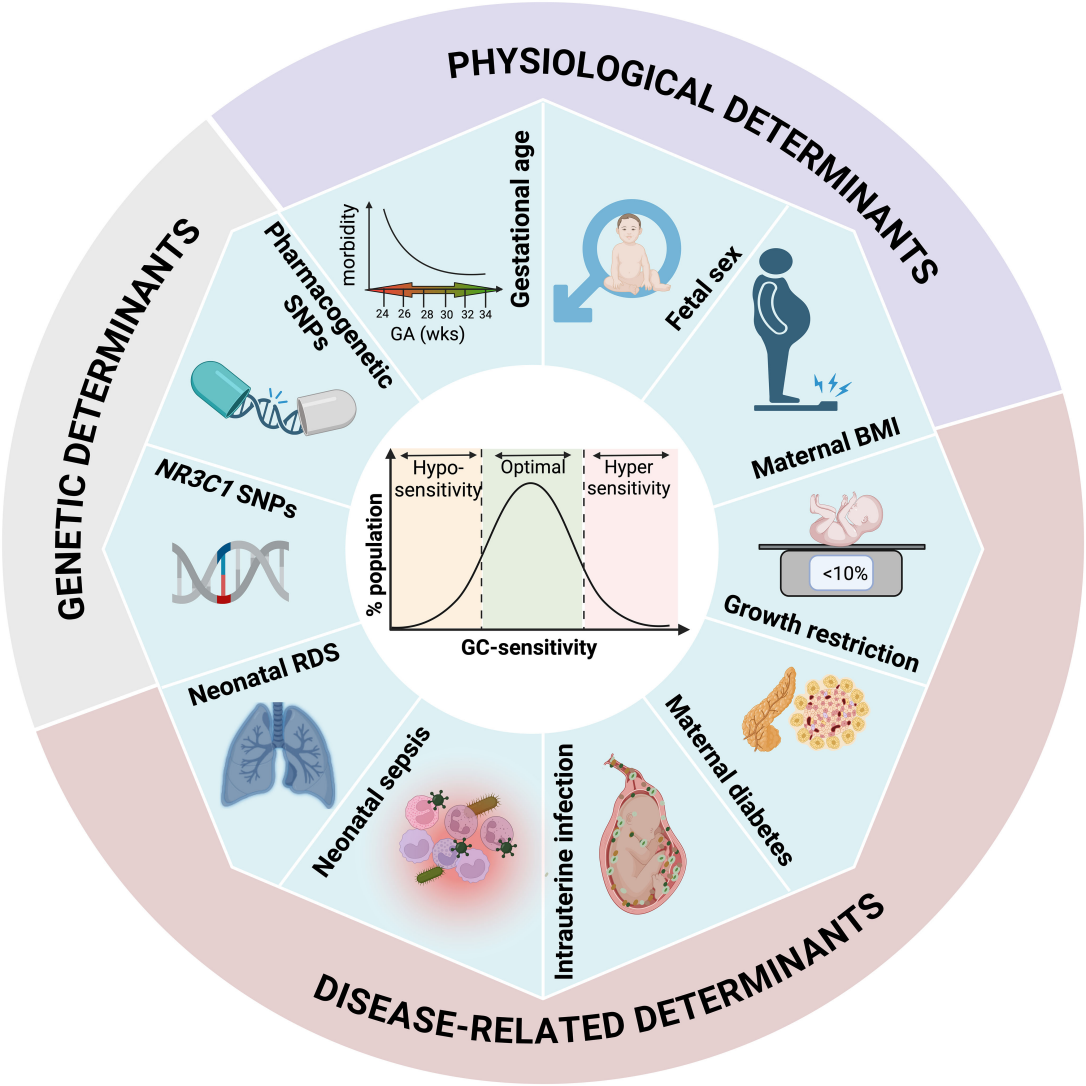
Endogenous determinants of glucocorticoid sensitivity in the preterm neonate. The *NR3C1 Bcl*1 variant is associated with an increased risk of BPD, while CYP3A, GSTP1, IPO13, and ABCB1 variants, involved in betamethasone metabolism and transport, are linked to a reduced risk of RDS. Physiological factors such as gestational age, fetal sex, and maternal BMI moderately alter HPA axis reactivity, tissue glucocorticoid bioavailability, and receptor homeostasis in the fetus and neonate. Importantly, perinatal complications such as IUGR amplify the effects of gestational age and fetal sex on perinatal glucocorticoid sensitivity. IUGR is associated with increased cortisol bioavailability without concurrent increases in lung maturation, suggesting tissue-specific resistance to cortisol. Diabetes is associated with reduced glucocorticoid sensitivity in adults, as well as in a preterm diabetic sheep model. Chorioamnionitis remains the only perinatal complication associated with increased perinatal glucocorticoid sensitivity, demonstrating concomitant increases in fetal cortisol bioavailability and lung maturation. In contrast, neonatal sepsis results in a blunted HPA axis and either high or low cortisol levels. Particularly, high cortisol levels were associated with circulatory collapse and death in preterm newborns, suggesting cardiovascular resistance to cortisol. Lastly, pulmonary and immune cell GR expression was reduced in severe RDS, though normal at birth, suggesting that GR downregulation plays a role in the progression of this disease. Ultimately, the interaction between these factors collectively modulates the glucocorticoid homeostasis in preterm neonates, influencing their vulnerability to morbidity.

Fetuses and neonates are not exempt from dysregulation of glucocorticoid sensitivity, primarily driven by intrauterine environmental cues. Perinatal exposure to supraphysiological glucocorticoid levels can disrupt normal developmental trajectories, potentially leading to postnatal diseases that often manifest in adulthood. Indeed, human and animal studies on the developmental origins of health and disease (DoHAD) have convincingly demonstrated that adverse intrauterine environments can lead to adult disease (215, 216). While DoHAD mechanisms are complex, earlier research suggested that intrauterine stressors increase fetal/placental cortisol bioavailability and program both the HPA axis and tissue GR homeostasis, thereby altering the offspring’s response to a postnatal stressor—an effect known as the “cortisol hypothesis”. Nevertheless, significant variability in dysregulation of glucocorticoid sensitivity has been observed in subjects exposed to similar intrauterine stressors. Researchers propose that differences in the timing and intensity of the stressor, along with genetic factors, contribute to this variability. Notably, much less is known about the short-term consequences of glucocorticoid homeostasis dysregulation in preterm neonates. In this section, we review human observational studies on the genetic, physiological, and disease-related determinants of perinatal glucocorticoid sensitivity (**Figure 4**). We also include *in vivo* and *in vitro* research that confirms human observations and explains the underlying molecular mechanisms. In this context, it is important to emphasize key limitations of the evidence presented below. First, observational studies on preterm newborns are confounded by multiple genetic and demographic variables, as well as known and unknown intrauterine stressors, including preterm birth itself. As a result, isolating the role of individual determinants of glucocorticoid sensitivity in preterm newborns is challenging. Second, most fetal and neonatal tissues of interest—such as the lung and brain—are inaccessible for functional assays. Third, the use of surrogate tissues such as PBMCs or placenta has not been thoroughly validated, limiting the ability to extrapolate findings to key organs like the lungs. Therefore, the discussion below should be interpreted with these limitations in mind.

### 5.1 Genetic determinants of perinatal glucocorticoid sensitivity

The association between common *NR3C1* gene variants and neonatal morbidity has been explored (**Table 1**). No correlation was found between the ER22/23K and N363S variants, which are associated with decreased and increased glucocorticoid sensitivity in adults, respectively, and neonatal morbidities (217–221). However, the N363S variant was associated with a reduced need for PCS, suggesting a protective effect (220). In contrast, findings on the association of the *Bcl*I variant with neonatal outcomes remain inconclusive. For instance, Bertalan et al. found no significant association between the *Bcl*I variant and neonatal morbidity (217), whereas Schreiner et al. reported an increased risk of BPD in *Bcl*I carriers (218). Remarkably, this effect was independent of other factors, although it had a smaller contribution to BPD risk than mechanical ventilation, gestational age, and SGA status (218). Furthermore, Haas et al. reported that maternal, but not neonatal, *Bcl*I variant is associated with reduced risk of neonatal RDS (222). Overall, the role of these variants in neonatal disease and potential underlying mechanisms remains poorly defined.

**Table 1:** Original human studies on the genetic determinants of perinatal glucocorticoid sensitivity.

Subject characteristics	Study design	Main findings	Reference
Total *n* = 125 Preterm neonates ■ACS treatment: 54.4% Avg GA: 31 weeks	-DNA source: neonatal -*NR3C1* SNPs: *Bcl*I, N363S, and ER22/23EK Association between genotypes and neonatal complications (RDS, BPD, NEC, IVH, PDA, and sepsis) and clinical parameters (birth weight) using one-way ANOVA-	- No significant associations of genetic variants with neonatal complications - *Bcl*I SNP associated with higher birth weight (*p* = 0.004), independent of ACS treatment	Bertalan et al. (2008) (217)
Total *n* = 10,490 -Preterm VLBW neonates ■ACS treatment: 88% Avg GA: 28.3 weeks■	-DNA source: neonatal -*NR3C1* SNPs: *Bcl*I, N363S, and ER22/23EK Association of genotypes with neonatal complications (RDS, BPD, NEC, IVH, PDA, sepsis, and MV) and clinical parameters (birthweight) was analyzed using logistic regression-	- *Bcl*I SNP associated with BPD (*p* = 0.015), PVL (*p* = 0.023), and surfactant treatment *(p* = 0.005) - *Bcl*I SNP associated with BPD in ACS-treated and Caucasian newborns - *Bcl*I SNP was an independent contributor to BPD (OR = 1.12, *p* = 0.013) - N363S SNP associated with MV (*p* = 0.032) and lower blood pressure on the first day of life (*p* = 0.008)	Schreiner et al. (2017) (218)
Total *n* = 41 - Preterm neonates ■ACS treatment: 51% GA range: 23–29 weeks■	-DNA source: neonatal -*NR3C1* SNPs: *Bcl*I, N363S, ER22/23EK, and 9β Association of genotypes with neonatal complications (RDS, BPD, NEC, PDA, ROP, sepsis, and MV) and clinical parameters (birthweight) was analyzed using logistic regression-	- No significant associations of genetic variants with neonatal diseases - *Bcl*I SNP is significantly associated with an increased rate of refractory hypotension (*p* < 0.001) - No carriers of 9β SNP	Ogasawara et al. (2018) (219)
Total *n* = 249 -Preterm neonates < 32 weeks GA ■ACS treatment: 21% Avg GA: 30 weeks■	-DNA source: neonatal *NR3C1* SNPs: N363S and -ER22/23EK Association of genotypes and neonatal (RDS, ICH, sepsis, postnatal glucocorticoid treatment) and long-term outcomes (1-month–19-year anthropometric and metabolic measurements) determined by linear regression analysis-	- No significant associations of genetic variants with neonatal diseases - N363S SNP associated with reduced use of postnatal glucocorticoid treatment (*p* = 0.02)	Finken et al. (2007) (220)
Total *n* = 294 - Preterm neonates ■ACS treatment: 21% Avg GA: 30 weeks■	-DNA source: neonatal *NR3C1* SNPs: N363S and -ER22/23EK Genotype association with short- (BPD, CPAP use) and long-term (1 month–19 years) outcomes (SOB, hay fever, asthma, wheezing, and eczema) using logistic regression analysis-	- No significant associations of *NR3C1* SNPs with neonatal diseases	Baas et al. (2021) (221)
Total *n* = 226 - Mothers *n* = 109 - Preterm neonates *n* = 117 ■ACS: 100% Avg GA: 32.2 weeks■	-DNA source: maternal and neonatal -73 SNPs in glucocorticoid-related genes, including drug-metabolizing enzymes (*CYP3A5* and *CYP3A7*), transport proteins (*IPO13* and *ABCB1*), signaling proteins (*ADCY9*), and hormone receptors (*CRHR1* and *NRC31*) Association of genotypes with RDS by logistic regression-	- Maternal SNPs associated with RDS: *CYP3A5* SNPs (OR = 1.63, *p* = 0.05); *NR3C1 Bcl*I SNP (OR = 0.28, *p* = 0.04) - Neonatal SNPs associated with RDS: *CYP3A7* SNPs (OR = 23.68, *p* = 0.03) *ADCY9* SNP (OR = 0.17, *p* = 0.02)	Haas et al. (2012) (222)
Total *n* = 226 - Mothers *n* = 109 - Preterm neonates *n* = 117 ■ACS: 100% Avg GA: 32.2 weeks■	-DNA source: maternal and neonatal -73 SNPs in glucocorticoid-related genes including drug-metabolizing enzymes (*CYP3A5* and *CYP3A7*), transport proteins (*IPO13* and *ABCB1*), signaling proteins (*ADCY9*) and hormone receptors (*CRHR1* and *NRC31*) Association of genotypes with neonatal respiratory outcomes other than RDS (MV, surfactant use, BPD) by logistic regression-	- Maternal SNPs : *ABCB1* SNPs negatively associated with MV (OR = 0.18, *p* = 0.05) *IPO13* SNPs positively associated with surfactant use (rs2428953 OR = 13.8 *p* = 0.01, rs2486014 OR = 23.8 *p* = 0.02) - Neonatal SNPs: *IPO13* SNPs negatively associated with BPD (OR = 0.01, *p* = 0.04)	Haas et al. (2013) (223)
Total *n* = 62 - Preterm neonates < 34 weeks GA ■ACS: 100% ■RDS Avg GA: 28.8 ■No-RDS Avg GA: 31.9	-DNA source: neonatal -*NR3C1* SNPs: *Bcl*I, N363S, and ER22/23EK -Placental transporter gene (*ABCB1*, *GSTP1*, *GSTM1*, *GSTT1*) SNPs Association of genotypes with neonatal RDS by logistic regression-	- *GSTP1* (I105V) negatively associated with RDS (OR = 0.154, *p* = 0.03)	Oretti et al. (2009) (228)

*ACS*, antenatal corticosteroid therapy; *ANOVA*, analysis of variance; *APGAR*, appearance, pulse, grimace, activity, respiration (used to assess newborn health); *Avg*, average; *BPD*, bronchopulmonary dysplasia; *CPAP*, continuous positive airway pressure; *GA*, gestational age; *ICH*, intracranial hemorrhage; *IVH*, intraventricular hemorrhage; *MV*, mechanical ventilation; *NEC*, necrotizing enterocolitis; *OR*, odds risk/ratio; *PDA*, patent ductus arteriosus; *PVL*, periventricular leukomalacia; *RDS*, respiratory distress syndrome; *ROP*, retinopathy of prematurity; *SNP*, single nucleotide polymorphism; *SOB*, shortness of breath; *VLBW*, very low birth weight.

In contrast to the *NR3C1* variants, polymorphisms in genes involved in glucocorticoid pharmacokinetics have demonstrated stronger associations with neonatal disease (**Table 1**). For instance, maternal *CYP3A5* variants, known to enhance CYP3A5 activity, were independently associated with an increased risk of RDS (223). Similarly, the fetal CYP3A7*1E variant, known to upregulate CYP3A expression, was also independently associated with an increased risk of RDS (223). Since CYP3A is the primary metabolizing enzyme of betamethasone, these polymorphisms likely accelerate maternal and fetal betamethasone inactivation, reducing its efficacy. In addition, maternal polymorphisms in the *ABCB1* gene, which encodes the efflux transporter P-glycoprotein, were associated with a reduced need for respiratory support (223). This effect may result from decreased pump activity in carriers of this variant (224), leading to increased betamethasone bioavailability. Interestingly, variants in the importin 13 (*IPO13*) gene exhibit opposing effects on neonatal outcomes depending on the carrier. In neonates, these variants reduce the risk of BPD, whereas in mothers, they increase the risk of surfactant use (223). Since importin 13 facilitates GR nuclear translocation, fetal *IPO13* polymorphisms may enhance glucocorticoid sensitivity and ACS efficacy by increasing GR nuclear presence. This aligns with previous findings showing that *IPO13* variants improve outcomes in asthmatic children receiving steroid treatment (225, 226). Neonates carrying the adenylate cyclase 9 (*ADCY9*) I772M variant have also shown reduced RDS risk (223). This variant has been linked to increased steroid response in asthmatics, although the protective mechanisms remain unclear (227). Finally, neonatal carriers of the glutathione *S*-transferase pi 1 (*GSTP1*) I105V polymorphism also exhibit a reduced risk of RDS (228). This variant decreases GST conjugating activity (229), potentially leading to reduced betamethasone conjugation and, thus, increased betamethasone bioavailability. However, this same variant is a risk factor for BPD, potentially due to reduced GSTP1-mediated protection against oxidative stress damage in the lungs (230).

### 5.2 Physiological determinants of perinatal glucocorticoid sensitivity

*Gestational age* is perhaps the most important determinant of fetal glucocorticoid sensitivity (**Table 2**). For example, extremely premature newborns (22–27 weeks of gestation) demonstrate a reduced risk of ACS-mediated neurodevelopmental impairment, while late preterm newborns (> 34 weeks of gestation) show the opposite effect (166). Similarly, ACS effects on HPA axis reactivity differ between preterm and term newborns: preterm newborns exhibit blunted HPA axis reactivity, whereas term newborns display heightened reactivity (178–181). Moreover, a recent study found significant differences in *NR3C1* methylation of promoter 1F, with extremely premature newborns showing higher methylation at birth, followed by decreased methylation at subsequent timepoints, compared to term newborns (231). Although the mechanisms remain uncertain, it is hypothesized that the term HPA axis is more susceptible to glucocorticoid-mediated downregulation of central GR expression, leading to impaired negative feedback regulation. Furthermore, *in vivo* studies suggest that gestational age modulates the maturational effects of ACS in the fetal lung (116). Interestingly, dexamethasone binding assays in autopsied fetal lung tissue have demonstrated relatively stable GR expression throughout gestation (104, 105). Additionally, GR protein levels were higher in lung homogenates from 15- to 28-week fetuses compared to infants and children (232). Concurrently, GRα mRNA expression in human fetal lung sections from subjects without apparent pulmonary disease remained consistent between 13 and 42 weeks of gestation but was higher than that of adults (233). Altogether, these findings suggest that gestational age does not significantly influence total pulmonary GR expression, although GR isoform expression patterns remain understudied. Parallel to the above findings, *in vitro* GR functional studies, using cord blood mononuclear cells (CBMCs), revealed that both preterm and term neonates display greater glucocorticoid sensitivity than children and adults (234–236). However, preterm newborns demonstrated lower endotoxin response and reduced basal dexamethasone sensitivity in IL-6 and IL-1β transrepression compared to healthy term newborns (235). Notably, the increased neonatal CBMC glucocorticoid sensitivity relative to adults was not attributed to differences in total GR levels or affinity (237–240). Lastly, a study on placental GR isoforms revealed that preterm newborns express higher GRα-C protein levels compared to term newborns (241). Alterations in placental GR expression were observed together with decreased placental 11β-HSD2 expression in preterm compared to term neonates (242). These data suggest increased placental glucocorticoid sensitivity in preterm compared to term neonates.

**Table 2:** Original human studies on the physiological determinants of perinatal glucocorticoid sensitivity.

Subject characteristics	Tissue	GC-sensitivity method	Main findings	Reference
Gestational age				
Total *n* = 90 Term neonates -ACS treated *n* = 30 ■Avg GA = 38 weeks -Non-ACS treated *n* = 60 Avg GA = 38.3 weeks■	Saliva at baseline, 20 min, and 40 min after heel-stick blood draw (stressor), performed 15–48 h after birth	Cortisol by EIA	-Larger cortisol stress response in ACS-treated neonates Higher cortisol response in ACS-treated neonates at GA 24–34 weeks versus > 34 weeks-	Davis et al. (2011) (178)
Total *n* = 80 Preterm neonates -Repeat-dose ACS *n* = 41 -Single-dose ACS *n* = 35 GA range = 23 to 32 weeks-	Cord serum and saliva collected at baseline, 30 min, 7 days, 14 days, and 21 days after heel-stick blood draw (stressor), on postnatal day 3	Cortisol by ELISA	-Lower salivary cortisol response poststress test (30 min) in the repeated-dose group than single-dose group Lower salivary cortisol levels on days 7, 14, and 21 in the repeat-dose ACS group compared to the single-dose group-	Ashwood et al. (2006) (179)
Total *n* = 103 - Extremely preterm *n* = 16 ■Avg GA = 26.4 weeks - Very preterm *n* = 42 ■Avg GA = 30.8 weeks - Healthy term *n* = 45 Avg GA = 40.1 weeks■	Plasma am and pm after vaccination at 4 months (stressor)	Cortisol by MS	- Blunted HPA axis reactivity in both preterm groups compared to term - Flattened diurnal cortisol slope among preterm neonates	Stoye et al. (2022) (180)
Total *n* = 181 Term and preterm neonates -ACS treated *n* = 110 -Non-ACS treated *n* = 71 -Avg GA = 36.6 weeks	Saliva collected at 1, 6, and 12 months postnatal at baseline and after psychological stressor protocol	Cortisol by ELISA	- Lower baseline cortisol in the ACS-treated group at 6 and 12 months -Blunted HPA axis response in ACS-treated neonates at 6 and 12 months	Weiss et al. (2023) (181)
Total *n* = 95 - Preterm *n* = 46 ■Median GA = 28.5 weeks - Term *n* = 49 Median GA = 39 weeks■	Genomic DNA from cord blood (day 0), peripheral blood (day 5), and buccal swabs (days 30 and 90)	DNA methylation of *NR3C1* promoter 1F by bisulfite-specific qPCR (EZ DNA methylation kit) Cortisol levels	- Higher methylation of three CpG sites in preterm neonates at day 0, followed by overall decreased methylation compared to term neonates - Higher cortisol levels—at day 5 only—in preterm compared to term - Increased cortisol levels with time in term, but not preterm, newborns	Chalfun et al. (2023) (231)
Total *n* = 33 - Hysterectomy *n* = 9 - Vaginal abortion *n* = 13 - Neonatal demise *n* = 11 GA range: 12–43 weeks■	Lung, autopsied fetuses or neonates, 0–29 h after death	GR number and affinity: radioactive DEX-binding assay	- No correlation between DEX binding and GA - DEX binding is detected as early as 12 weeks of gestation and is highest in the lung compared to other organs (liver, kidney)	Ballard and Ballard (1974) (104)
Total *n* = 15 - 9 fetuses, abortions ■GA range: 15–28 weeks - 6 neonates and children ■Age: 2 months–9 years	Lung, autopsied (fetuses), or lobar resections (children)	GR number and affinity: radioactive DEX-binding assay	- No correlation between DEX binding and GA - DEX binding in children is significantly lower than in fetuses (*p* < 0.001)	Labbé et al. (1990) (232)
Total *n* = 25 - Fetuses without pulmonary disease (*n* = 20, 5 per lung developmental stage: pseudoglandular, canalicular, saccular, and alveolar) - Adults *n* = 5 Age: 25–49 years■	Fetal lung, autopsied within 24 h of death Adult lung resections	GR mRNA and protein levels by RT-PCR and IHC	- No correlation between GR mRNA levels and GA - Increased GR mRNA levels at the canalicular stage, remaining high throughout gestation - GR mRNA levels are lower in adults than in fetuses - GR protein detected as early as 13 weeks of gestation	Rajatapiti et al. (2005) (233)
Total *n* = 86 - Preterm *n* = 26 ■ACS: 0% ■Avg GA: 29 weeks - Healthy term *n* = 36 ■Avg GA not reported - Healthy adult *n* = 24 Avg age: 37.2 years	CBMCs (neonates) PBMCs (adults)	Cell stimulation with PHA ± DEX (10^−8^ to 10^−5^M) × 24 h; media tested for IL-2 and IL-3 activity by cell proliferation	- Similar preterm and term neonate DEX inhibition of IL-2 and IL-3 - Higher DEX inhibition of IL-2/IL-3 in neonates (preterm and term) than in adults	Bessler et al. (1996) (234)
Total *n* = 72 - Preterm *n* = 20 ■ACS: 0% ■Avg GA: 29.1 weeks - Healthy term *n* = 22 ■Avg GA not reported - Healthy adult *n* = 30 Avg age: 37.2 years	CBMCs (neonates) PBMCs (adults)	Cell stimulation with LPS ± DEX (10^−8^ to 10^−5^ M) for 24 h; media tested for IL-1β, TNFα, and IL-6 by ELISA	- Decreased DEX inhibition of basal IL-1β and IL-6 in preterm compared to term/adult - Lower LPS induction of IL1β, TNF-α, and IL-6 in preterm than term/adult - Higher DEX inhibition of LPS-induced IL-1β, TNF-α, and IL-6 in neonates (preterm and term) than in adults	Bessler et al. (1999) (235)
Total *n* = 61 - Preterm *n* = 20 ■Avg GA: 29 weeks ■ACS: 0% - Healthy term *n* = 20 ■Avg GA not reported -Healthy adults *n* = 21 ■Avg age: 37 years	CBMCs (neonates) PBMCs (adults)	Cell stimulation with LPS ± DEX (10^−8^ to 10^−5^ M) for 24 h; media tested for IL-10 and IL-12p40 by ELISA	- Higher LPS-stimulated IL-10 production in adults compared to term and preterm - DEX inhibition of IL-10 production in adult cells only - Higher basal IL-12 in term CBMCs than preterm CBMCs and adult PBMCs - Higher DEX-mediated inhibition of IL12 in term and preterm CBMCs compared to adult PBMCs	Bessler et al. (2001) (236)
Total *n* = 50 - Preterm *n* = 30 ■Avg GA: 32.42 weeks - Healthy term *n* = 20 Avg GA: 39.6 weeks■	- PBMCs from preterm neonates (2–3 days old) - CBMCs or PBMCs from term neonates (at birth)	GR number and affinity: radioactive DEX-binding assay	- No correlation between DEX binding and GA - Similar DEX binding between preterm and term groups	Kerepesi and Arányi (1985) (237)
Total *n* = 35 - Preterm *n* = 20 ■Avg GA: 30.62 weeks - Healthy term *n* = 15 Avg GA: not reported■	CBMCs	GR number and affinity: radioactive DEX-binding assay	- Similar DEX binding in term compared to preterm samples	Vlugt et al. (1997) (238)
Total *n* = 131 - Preterm *n* = 54 ■Avg GA: 29.2 weeks - Term *n* = 77 Avg GA: 38.8 weeks■	Whole blood from UC vessels or from a neonate’s vein/artery at NICU day 0	GRα and GRβ mRNA isoforms by qPCR	- Higher GRβ at birth in preterm than term neonates	Go et al. (2013) (239)
Total *n* = 75 - VLBW preterm *n* = 43 ■Avg GA: 27.6 weeks - Term neonates *n* = 32 GA range: 37–42 weeks■	CBMCs (day 0) and PBMCs at days 4–7 (only preterm neonates)	GRα and GRβ mRNA isoforms by RT-qPCR	- Higher GRα and GRβ levels at birth (day 0) compared to days 4–7 in VLBW preterm neonates - Lower GRα and GRα/GRβ ratio in VLBW preterm than term neonates	Yamamoto et al. (2022) (240)
Total *n* = 110 - Healthy term *n* = 55 ■Avg GA: 39.4 weeks - Preterm *n* = 55 ■ACS: 47.3% Avg GA: 33.3 weeks■	Placenta	GR protein isoform levels by Western blot	- Higher GRα-C protein levels in preterm than term placenta	Saif et al. (2015) (241)
Total *n* = 244 - Preterm *n* = 104 ■14 GA 24–28 weeks ■25 GA 28–32 weeks ■64 GA 32–36 weeks - Healthy term *n* = 140 Avg GA not reported■	Placenta	11β-HSD2 mRNA by RT-qPCR	- Decreased 11β-HSD2 in preterm neonates at GA 28–36 compared with term - Similar 11β-HSD2 expression in preterm neonates at GA 24–28 weeks compared to term - Similar 11β-HSD2 expression in male and female neonates	Demendi et al. (2012) (242)
Fetal sex				
Total *n* = 34 All healthy term ■Vaginal: 53% ■Male: 50% GA not reported■	Placenta	GR mRNA isoforms (5′UTR and 3′UTR isoforms) by RT-qPCR	- Higher GR1A3 in male than female neonates - Lower GR-P mRNA levels in vaginal delivery samples	Johnson et al. (2008) (243)
Total *n* = 135 - Healthy term *n* = 53 ■Male: 45% - Asthmatic term *n* = 82 ■Male: 50% Avg GA: 39.5 weeks■	Placenta	GR protein isoform levels by Western blot, cortisol levels by ELISA	- Positive correlation of cortisol levels with placental GRβ protein in healthy male neonates only - Higher GRβ protein in male placentas of asthmatics than in healthy male placentas - Higher GRβ and GRα-D1 protein in placentas of SGA than AGA	Saif et al. (2014) (244)
Total *n* = 174 -Term ■Male *n* = 92 - 22 control - 20 mild asthma - 18 severe asthma ■Female *n* = 59 - 11 control - 16 mild asthma - 29 severe asthma	UC plasma + placenta	Cortisol by RIA and placental GR mRNA expression by RT-qPCR	- Higher UC cortisol levels in term female neonates exposed to moderate-to-severe asthma compared with control female and male neonates - Lower placental total GR protein levels in female neonates (all 3 groups) compared with male neonates	Hodyl et al. (2010) (245)
Total *n* = 182 - All term ■Male *n* = 92 - 23 control - 24 asthma without iCS - 45 asthma with iCS ■Female *n* = 59 - 21 control - 22 asthma without iCS - 47 asthma with iCS	UC vein plasma + placenta	Cortisol by RIA and placental 11β-HSD2 activity by radioactive assay	- Lower birthweight in association with lower placental 11β-HSD2 activity in female newborns of asthmatic mothers without iCS compared to other groups - No differences among male newborns from different groups - No correlation of UC cortisol levels with other parameters	Murphy et al. (2003) (246)
Total *n* = 174 - Term ■Lean BMI *n* = 43 - 14 male neonates - 29 female neonates ■Morbid obesity *n* = 50 - 30 male neonates - 20 female neonates	Maternal serum, placenta, survey (emotional distress)	Cortisol by RIA and placental glucocorticoid bioavailability-relevant gene expression by RT-qPCR	- Reduced placental expression of *ABCB1*, *ABCG2*, *11β-HSD2*, and GRα in female neonates with maternal distress; no effect in male neonates	Mina et al. (2015) (247)
Total *n* = 114 - Preterm, 100% ACS ■Male *n* = 55 ■Female *n* = 59 ■Avg GA: not stated ■Birthweight at ACS 2–10 days before delivery - 751–1,250 g *n* =20 - 1,251–1,750 g *n* = 36	UC serum	Betamethasone levels by radioreceptor assay Cortisol and DHEAS by RIAs	- Lower RDS rate in females than males in the 1,251–1,750 g birthweight category with ACS exposure 2–10 days before delivery - No effect of fetal sex on RDS, cortisol, or betamethasone levels when ACS exposure interval was < 2 days from birth or in the 750–1,250 g birthweight category - Sex differences are not associated with betamethasone or cortisol levels	Ballard et al. (1980) (248)
Total *n* = 110 - Healthy term *n* = 55 ■Male samples: 44% ■Avg GA: 39.4 weeks - Preterm *n* = 55 ■ACS: 47.3% ■Male samples: 54.5% Avg GA: 33.3 weeks■	Placenta	GR protein isoform levels by Western blot	- Higher GRα-D2 protein isoform in preterm male compared to preterm female samples - Lower GR A, GRα-D1, and GRα-D2 in SGA than AGA in female preterm samples; no effect in male preterm samples	Saif et al. (2015) (241)
Total *n* = 50 - Preterm *n* = 30 ■Avg GA: 32.42 weeks Male samples: 47%■	PBMCs from preterm neonates (2–3 days old)	GR number and affinity: radioactive DEX-binding assay	- DEX binding did not correlate with fetal sex or birthweight in the premature neonate group	Kerepesi and Arányi (1985) (237)
Total *n* = 131 - Preterm *n* = 54 ■SGA: 22.2% ■Male samples: 57.4% ■ACS: 40.7% ■Avg GA: 29.2 weeks - Term *n* = 77 ■SGA: 13% ■Male samples: 48% Avg GA: 38.8 weeks■	Whole blood from UC vessels, or from a neonate’s vein/artery at NICU day 0	GRα and GRβ mRNA isoforms by qPCR	- No effect of fetal sex, ACS, or mode of delivery on GR expression - Higher GRβ and lower GRα/GRβ in SGA-preterm compared to AGA-preterm neonates - No effect of birthweight on GR levels in term neonates	Go et al. (2013) (239)
Total *n* = 75 - VLBW preterm *n* = 43 ■Male samples: 53.5% ■SGA: 40% ■ACS: 86% Avg GA: 27.6 weeks■	CBMCs (day 0) and PBMCs at days 4–7 (only from preterm neonates)	GRα and GRβ mRNA isoforms by RT-qPCR	- No effect of fetal sex, birthweight, ACS, or mode of delivery on GR mRNA levels	Yamamoto et al. (2022) (240)
Total *n* = 172 All healthy term ■Male samples: 50% ■Avg birthweight: 2,951 g GA range: 37–42 weeks■	CBMCs and plasma	GRα and GRβ mRNA levels by RT-qPCR, plasma cortisol assay by ELISA	- No effect of fetal sex, fetal number, birthweight, or mode of delivery on GR mRNA levels - Cortisol levels did not correlate with fetal sex, fetal number, birthweight, or mode of delivery	Imamura et al. (2011) (250)
Total *n* = 905 - Preterm neonates A. ELGAN cohort (*n* = 365, Avg GA 26.1 weeks) ■BDP: 53.7% ■ACS: 91% B. NOVI cohort (*n* = 538, Avg GA 27 weeks) ■BDP: 51.5% ACS: 89%■	Genomic DNA from: A. Blood spot around birth (ELGAN) B. Buccal swabs prior to discharge (NOVI)	DNA methylation data obtained using Illumina Methylation EPIC microarray	- NOVI cohort only (at time of discharge, after BPD development): 10 differentially methylated CpGs in 4 glucocorticoid-related genes (*CRHR1*, *HSP90AA1*, *NR3C1*, *NR3C2*) in ACS versus no-ACS exposure in male compared to female samples	Hodge et al. (2024) (251)
Maternal BMI				
Total *n* = 78 ■Pregravid BMI range: 17.4–41 kg/m^2^ Avg GA at sampling: 15.9 weeks■	Amniotic fluid from amniocentesis in the second trimester (~ 15 weeks GA)	Free cortisol and free cortisone measured by MS to assess11β-HSD2 activity indirectly	- Fetoplacental 11β-HSD2 activity (ratio of cortisone to cortisol) negatively correlated with maternal BMI	Lamadé et al. (2021) (254)
Total *n* = 111 - All term neonates ■GA: 37–41 weeks Pregravid BMI IQR: ■19.5–31.7 kg/m^2^	Placenta and cord blood	Cortisol by ELISA Placental 11β-HSD2 activity by radioactive cortisol to cortisone conversion	- Placental 11β-HSD2 activity negatively correlated with maternal BMI (*r* = − 0.198, *p* = 0.037) - Cord blood cortisol did not correlate with BMI	Słabuszweska-Jóżwiak et al. (2020) (255)
Total *n* = 331 - All term pregnancies ■Lean *n* = 132 ■Avg GA: 39.4 weeks Overweight *n* = 56 ■Avg GA: 39.5 weeks Obese *n* = 64 ■Avg GA: 39.8 weeks GDM *n* = 79 Avg GA: 39.2 weeks■	Maternal peripheral blood (collected at 24 and 34 weeks and delivery) and cord blood	Cortisol assay by an unreported method	- Lower maternal cortisol levels at delivery in severely obese compared to lean mothers - No effect of maternal BMI on maternal cortisol levels at 24 and 34 weeks of gestation - No effect of maternal BMI on cord blood cortisol levels	Berglund et al. (2016) (256)
Total *n* = 259 - All term pregnancies ■Lean *n* = 102 Avg GA: 39.1 weeks ■Overweight *n* = 79 Avg GA: 39.3 weeks ■Obese *n* = 45 Avg GA: 39.3 weeks ■Severely obese* *n* = 33 Avg GA: 39.4 weeks	Maternal peripheral blood (collected at delivery) and cord blood	Glucocorticoid levels (cortisol, cortisone, corticosterone, and 11-dehydrocorticosterone) by LC-MS	- Negative correlation between maternal BMI and maternal cortisol, corticosterone, and 11-dehydrocorticosterone - Positive correlation between maternal and umbilical cord blood levels of glucocorticoids and their metabolites - No correlation between maternal BMI and cord blood glucocorticoid levels	Stirrat et al. (2017) (257)
Total *n* = 42 All healthy term ■Maternal BMI: -DEX sensitive: 25.4 kg/m^2^ -DEX resistant: 22.1 kg/m^2^ Male samples: 50%■	HUVEC and cord blood	DEX treatment (10^−8^ to 10^−6^M) for 24 h to test regulation of vascular genes and GRα expression by RT-qPCR and Western blot	- Positive correlation between maternal prepregnancy and *in vitro* HUVEC-DEX sensitivity (*p* = 0.046) - Higher basal and stimulated GR protein and lower BAG1 E3 ubiquitin ligase expression in DEX-sensitive compared to DEX-resistant HUVECs	Mata-Greenwood et al. (2013) (259)
Total *n* = 25 All healthy term ■Maternal BMI: -DEX sensitive: 27.2 kg/m^2^ -DEX resistant: 22 kg/m^2^ Male samples: 60%■	HUVEC	DEX treatment (10^−8^ to 10^−6^M) for 24 h; GR mRNA isoform expression by RT-qPCR and promoter methylation by MeDIP assay and bisulfite sequencing	- Positive correlation between maternal BMI and *in vitro* DEX sensitivity (*p* = 0.038) - Lower basal expression of GR-1C and GR-1D and higher 1F isoforms in DEX-sensitive cells - Higher promoter 1D methylation and lower promoter 1F methylation in DEX-sensitive cells	Mata-Greenwood et al. (2015) (262)
Cortisol levels and ACS exposure				
Total *n* = 35 - Preterm *n* = 20 ■ACS: 65% Avg GA: 30.62■	CBMCs	GR number and affinity: radioactive DEX-binding assay	- No effect of ACS on DEX-binding levels	Vlugt et al. (1997) (238)
Total *n* = 131 - Preterm *n* = 54 ■ACS: 40.7% Avg GA: 29.2 weeks■	Whole blood from UC vessels or from a neonate’s vein/artery at NICU day 0	GRα and GRβ mRNA isoforms by qPCR	- No effect of ACS on GR mRNA levels	Go et al. (2013) (239)
Total *n* = 75 - VLBW preterm *n* = 43 ■ACS: 86% Avg GA: 27.6 weeks■	CBMCs (day 0) and PBMCs (days 4–7)	GRα and GRβ mRNA isoforms by RT-qPCR	- No effect of ACS on GR mRNA levels	Yamamoto et al. (2022) (240)
Total *n* = 172 All healthy term ■Male samples: 50% GA range: 37–42 weeks■	CBMCs	GRα and GRβ mRNA levels by RT-qPCR, plasma cortisol assay by ELISA	- No correlation between cortisol levels and GRα/GRβ mRNA expression	Imamura et al. (2011) (250)
Total *n* = 135 - Healthy term *n* = 53 ■Male samples: 45% - Asthmatic term *n* = 82 Male samples: 50%■	Placenta	GR protein isoform levels by Western blot, cortisol levels by ELISA	- Positive correlation between cortisol levels and GRβ protein in healthy male placentas only - No correlation between cortisol and GR expression in female or asthmatic samples	Saif et al. (2014) (244)
Total *n* = 110 - Preterm *n* = 55 ■ACS: 47.3% ■Male samples: 54.5% Avg GA: 33.3 weeks■	Placenta	GR protein isoform levels by Western blot	- No effect of ACS on GR protein levels	Saif et al. (2015) (241)
Total *n* = 42 All healthy term Male samples: 50%■	HUVEC UC blood	DEX treatment (10^−8^ to 10^−6^ M) to test gene expression and GR function, cortisol assay by ELISA	- No association of cord blood cortisol levels with HUVEC DEX sensitivity, or HUVEC-GR expression	Mata-Greenwood et al. (2013) (259)
Total *n* = 51 - Late preterm ■ACS: 53% GA range: 34–37 weeks	Cord blood, CBMCs, and isolated UC CD4^+^ T cells	Flow cytometry of immune cell frequencies Promoter 1F *NR3C1* methylation in UC CD4^+^ T cells by Illumina Methylation 450 BeadChip qPCR of GR 5′UTR mRNA isoforms	- ACS treatment is associated with increased granulocyte and decreased lymphocyte cord blood populations - No effect of ACS on *NR3C1* promoter methylation - No effect of ACS on various GR 5′UTR mRNA isoform levels	Carpenter et al. (2022) (263)
Total *n* = 905 - Preterm neonates A. ELGAN cohort (*n* = 365, Avg GA 26.1 weeks) ■BDP: 53.7% ■ACS: 91% B. NOVI cohort (*n* = 538, Avg GA 27 weeks) ■BDP: 51.5% ACS: 89%■	Genomic DNA from: A. Blood spot around birth (ELGAN) B. Buccal swabs prior to discharge (NOVI)	DNA methylation data obtained using Illumina Methylation EPIC microarray	NOVI cohort only (after BPD development): - ACS exposure correlated with differential methylation in HPA axis-related genes, including *FKBP5*, *NR3C1*, *NR3C2*, *CRHR1*, *POMC*, and *HSP90AA* - The most significant differentially methylated site in the body of *NR3C1*	Hodge et al. (2024) (251)
Total *n* = 120 All preterm ■ACS: 41.6% ■Birthweight: ≤ 1,500 g ■Avg GA: 27.6 weeks Respiratory support: 100%■	PBMCs (collected on days 5 and 28 after delivery)	Secondary analysis of a Microarray transcriptomic study	- Day 5: 13 DEGs between ACS-treated and control groups ■4 upregulated genes related to cancer/inflammation ■6 downregulated genes were Y-linked and related to male infertility and cell differentiation ■3 downregulated genes related to preeclampsia and oxidative stress - Day 28: only 1 DEG	Saugstad et al. (2013) (264)

*ACS*, antenatal corticosteroids; *AGA*, average for gestational age; *Avg*, average; *BMI*, body mass index; *BMZ*, betamethasone; *CBMC*, cord blood mononuclear cells; *DEX*, dexamethasone; *DEG*, differentially expressed gene; *GA*, gestational age; *GDM*, gestational diabetes; *GR*, glucocorticoid receptor; *11β-HSD*, 11beta-hydroxysteroid dehydrogenase; *iCS*, inhaled corticosteroids; *IQR*, interquartile range; *LC-MS*, liquid chromatography mass spectrometry; *LPS*, lipopolysaccharide; *MeDIP*, methyl-DNA immunoprecipitation; *MR*, mineralocorticoid receptor; *PBMC*, peripheral blood mononuclear cells; *PHA*, phytohemagglutinin; *RDS*, respiratory distress syndrome; *SGA*, small for gestational age; *UC*, umbilical cord; *UTR*, untranslated region; *VLBW*, very low birth weight.

*Fetal sex* has been shown to play a role in regulating the HPA axis and tissue GR homeostasis in healthy term neonates (**Table 2**). For instance, male subjects showed a positive association between cortisol levels and placental GRβ protein, along with higher placental GR1A3 mRNA compared to females (243, 244). The GR1A3 transcript isoform is predominantly expressed in immune cells, suggesting differential regulation of placental leukocyte-GR expression in male compared to female subjects. Furthermore, the correlation between placental GRβ protein expression and fetal cortisol levels suggests that male subjects are more sensitive to cortisol-mediated GRβ regulation than female subjects. Sexual dimorphism was accentuated in the presence of a stressor, such as maternal asthma. For instance, moderate to severe maternal asthma was associated with reduced birthweights in term female neonates only, together with higher fetal cortisol levels and decreased placental 11β-HSD2 and GRβ expression (244–246). Similarly, maternal psychosocial stress was linked to decreased placental 11β-HSD2 and GRα expression in term female subjects only (247). Prematurity is another important stressor that amplifies sexual dimorphism in perinatal glucocorticoid sensitivity. An earlier study found lower RDS rates in ACS-exposed preterm female compared to male subjects matched for birth weight (248). This sexual dimorphism was confirmed in ovine models, with preterm female lambs showing improved respiratory outcomes compared to male subjects (249), although the underlying mechanisms remain elusive. Prematurity and fetal sex also interacted in the regulation of placental GR expression, with preterm males expressing higher GRα-D2 protein levels than preterm female subjects (241). Moreover, an interaction between prematurity, birthweight, and fetal sex was observed: in preterm female subjects, placental GRα-D1 and GRα-D2 protein levels were lower in small-for-gestational-age (SGA) compared to average-for-gestational-age (AGA) neonates, with no effect of birthweight in preterm male subjects (241). Interestingly, no sexual dimorphism in GR transcript variants was found in CBMCs from preterm/term neonates (237–240, 250), further emphasizing glucocorticoid tissue-specific effects. Similarly, there were no differences in HPA gene methylation in very preterm newborns at birth; yet after development of BPD, male neonates presented 10 differentially methylated sites in four genes—including *NR3C1*—with ACS exposure compared with female neonates (251). Altogether, these findings suggest that intrauterine stressors elicit sexually dimorphic adaptations in glucocorticoid sensitivity in key tissues such as the placenta, with female neonates showing upregulation of highly sensitive GR isoforms, potentially leading to increased sensitivity to glucocorticoid-mediated growth inhibition.

DoHAD animal studies have demonstrated a causal regulatory effect of maternal nutrition on the fetal/neonatal HPA axis and tissue glucocorticoid sensitivity (252). In contrast, human studies have been restricted to the association between maternal BMI, as a proxy for nutritional status, and neonatal glucocorticoid sensitivity (253) (**Table 2**). In this respect, underweight mothers with a BMI < 18 kg/m^2^ are at risk of presenting IUGR, whereas mothers with a BMI > 30 kg/m^2^ are at risk of macrosomia and gestational diabetes (252). Importantly, these pregnancy complications encompass a range of pathologies with different etiologies; thus, their role in perinatal glucocorticoid sensitivity will be discussed in the sections below. Increased interest in the role of maternal obesity in maternal–neonatal outcomes has sparked research into uncomplicated pregnancies across various maternal BMI ranges. Maternal obesity, which complicates ~ 40% of all pregnancies in the USA, has been associated with reduced placental 11β-HSD2 activity, with no effect on cord blood cortisol levels (254, 255). Furthermore, severely obese pregnant women exhibited reduced cortisol levels compared to healthy controls (256, 257), suggesting a role for decreased cortisol bioavailability in maternal obesity-induced fetal macrosomia (258). In addition, research on human umbilical vein endothelial cells (HUVEC) found a positive association between maternal BMI and GR expression, as well as *in vitro* dexamethasone sensitivity (259). The effect of maternal obesity on fetal endothelial GR expression was confirmed in a diet-induced maternal obesity ovine model (260), which also showed GR upregulation in the fetal heart (261). Increased HUVEC-GR expression was primarily due to reduced GR proteasomal degradation (259). Furthermore, differential *NR3C1* gene methylation at nondominant promoters 1D and 1F was associated with glucocorticoid sensitivity (262), highlighting the important role of maternal obesity in the epigenetic remodeling of fetal tissues.

Finally, fetal circulating cortisol levels and ACS exposure were not associated with CBMC-, placenta-, or HUVEC-GR expression in neonates (**Table 2**). Furthermore, ACS exposure did not affect cord blood CD4^+^ T-cell methylation levels of the *NR3C1* promoter or GR 5′UTR mRNA isoform levels in late-preterm neonates (263). Similarly, ACS exposure did not affect methylation status of HPA genes—including *NR3C1*—at birth, but significantly altered 10 CpG sites at discharge time (251). Finally, a microarray study on ACS transcriptional profiles in preterm neonatal PBMCs identified only 13 differentially expressed genes in samples from postnatal day 5, and just one differentially expressed gene in samples collected at postnatal day 28 (264). Overall, these studies suggest that ACS exposure has a minor and transient effect on GR expression and function in most fetal/neonatal tissues studied to date. This uncoupling of circulating glucocorticoid levels from tissue GR expression contrasts with the well-known *in vitro* effects of glucocorticoids in downregulating their own receptor (20, 62). It can be proposed that multiple redundant mechanisms of tissue GR regulation maintain a relatively stable expression and function *in vivo* under mild-to-moderate stress. As discussed in the following paragraphs, perinatal complications can further dysregulate glucocorticoid homeostasis.

### 5.3 Disease-specific determinants of perinatal glucocorticoid sensitivity

Multiple perinatal complications have been associated with fetal/neonatal dysregulation of the HPA axis, cortisol metabolism, and tissue GR expression and function (**Table 3**). Furthermore, there is concern regarding the proper use of ACS in special populations, such as those with IUGR, maternal diabetes, and chorioamnionitis, due to their independent contributions to neonatal morbidity and their potential to decrease the therapeutic index of ACS (265, 266). Currently, it remains unclear whether intrauterine stressors affect fetal predisposition to prematurity-related diseases via unchecked inflammatory signaling, dysregulated glucocorticoid sensitivity, or a combination of both. Notably, conflicting and inconsistent results have been observed, possibly due to the limited analysis of only a few glucocorticoid-related parameters in a heterogeneous population of preterm newborns. In this subsection, we summarize and discuss original research studies that illustrate the role of key perinatal complications on fetal/neonatal glucocorticoid homeostasis. Insights from pediatric and adult studies, as well as animal and *in vitro* research, are included to broaden understanding of concepts and mechanisms, with careful consideration of the limitations previously mentioned.

**Table 3:** Original human studies on the disease-related determinants of perinatal glucocorticoid sensitivity.

Subject characteristics	Tissue	GC-sensitivity method	Main results	Reference
Intrauterine growth restriction				
Total *n* = 65 - All preterm neonates ■SGA: 30.7% ■AGA: 69.3%, GA-matched ■Avg GA: 32.3 weeks	Saliva, postnatal days 0–92, collected at 10 am and 7 pm	- Cortisol by ELISA (groups; postnatal days 0–5, 6–14, and > 14)	- Higher 0–5-day neonatal cortisol levels in SGA neonates; no sexual dimorphism - Lower cortisol levels in SGA after day 14	Iwata et al. (2019) (269)
Total *n* = 61 - All preterm neonates ■SGA: 39.3% ■Controls: 60.7% GA-matched GA range: 24–36 weeks■	UC vein (at birth) and venipuncture (at 1 month) collected at 9 am	- Cortisol by electrochemiluminescence immunoassay	- Higher cortisol levels in SGA at birth - Similar cortisol levels in SGA and AGA neonates at 1 month	Aoki et al. (2022) (270)
Total *n* = 102 - All preterm neonates ■SGA: 41/102 GA range: 18–36 weeks■	Cord blood by cordocentesis	- Cortisol and ACTH by RIA	- Higher cortisol and lower ACTH levels in SGA	Economides et al. (1988) (271)
Total *n* = 56 - Preterm (28/56) and term (28/56) ■SGA: 50% in both groups ■AGA: 50%, GA-matched to SGA GA range: 31–43 weeks■	Cord blood	- Cortisol, ACTH, CRH, and DHEAS by RIA	- Higher CRH and ACTH levels in SGA - Nonsignificant higher cortisol levels in SGA - Lower DHEAS levels in SGA	Goland et al. (1993) (272)
Total *n* = 139 - All term neonates ■IUGR: 48.9%, GA avg 38.7 Controls: GA-matched, GA avg: 38.5■	Cord blood	- Cortisol by EIA, neonates (groups: 3 tiers of cortisol levels)	- Lower cortisol levels in IUGR neonates	Strinic et al. (2007) (273)
Total *n* = 71 - All term neonates ■IUGR: 31/71% ■Controls: GA-matched GA avg: 39.3 weeks■	Amniotic fluid after rupture of membranes and cord blood	- Cortisol by immunofluorometric assay	- Lower amniotic cortisol in IUGR neonates - Similar cord blood cortisol levels in IUGR and AGA neonates	Nieto-Diaz et al. (1996) (274)
Total *n* = 45 - All term neonates ■SGA: 75% ■Controls: GA-matched GA range: 38–42 weeks■	Cord blood	- Cortisol by RIA	- Lower cortisol levels in IUGR neonates	Cianfarani et al. (1998) (145)
Total *n* = 43 - All preterm neonates - SGA: 23.3%, GA-matched - GA range 25–32 weeks	Serum, before, 30 min, and 60 min after ACTH test, 5–12 days of age in clinically stable neonates	Cortisol and -18-dihydroxy-progesterone (17-OHP), by ELISA	- Blunted cortisol response to ACTH in SGA neonates, corrected after GA, fetal sex, and parity	Bolt et al. (2002) (276)
Total *n* = 58 - Late preterm–term neonates ■SGA: 31%, GA-matched GA range: 34–42■	Saliva before and after heel prick, 3–4 days postnatally	- HPA axis stress test (heel-prick) reactivity - Cortisol and cortisone by LC/MS	- Blunted HPA axis reactivity in SGA neonates	Schäffer et al. (2009) (277)
Total *n* = 76 - All preterm ■IUGR preterm *n* = 12: GA range: 25–36 weeks ■AGA preterm *n* = 14, GA range: 27–36 weeks AGA term *n* = 50, GA range: 39–40 weeks■	Placenta	-Cortisol and cortisone by RIA 11β-HSD2 activity assay based on cortisol to cortisone conversion-	- Reduced 11β-HSD2 activity in IUGR neonates - Inverse correlation between 11β-HSD2 activity and GA in IUGR neonates - Positive correlation between 11β-HSD2 activity and GA in AGA preterm and term	Shams et al. (1998) (278)
Total *n* = 24 - All term IUGR *n* = 12, ■Avg GA: 37.8 weeks AGA *n* = 12, Avg GA: 39.2 weeks■	Placenta UC artery blood	-Arterial cortisol and cortisone by RIA -11β-HSD2 mRNA levels by qPCR 11β-HSD2 activity by conversion of [H]^3^-cortisol to cortisone-	- Lower 11β-HSD2 activity and mRNA levels in IUGR placentas - Higher UC arterial cortisol/corticosterone ratio in IUGR	Dy et al. (2008) (279)
Total *n* = 241 - Preterm and term ■IUGR: 41.9% Avg GA: 37.5 weeks■	Placenta	- 11β-HSD2 expression by real-time qPCR	- Lower 11β-HSD2 expression in IUGR placentas at GA > 33 weeks - No differences in 11β-HSD2 expression between IUGR and AGA at GA < 33 weeks	Börzsönyi et al. (2012) (280)
Total *n* = 86 - Term and preterm IUGR *n* = 19, ■ GA range: 25–38 weeks Controls: *n* = 26, GA range: 27–40 weeks■	Placenta	-11β-HSD2 expression by qPCR -Mutations and imprinting by sequencing assays	- Reduced expression of 11β-HSD2 mRNA in IUGR - No mutations or imprinting in the *11β-HSD2* gene	McTernan et al. (2001) (281)
Total *n* = 44 - Term and late-preterm IUGR *n* = 22, ■Avg GA: 37.5 weeks AGA *n* = 22, Avg GA: 39.2 weeks■	Placenta	- *HSD11B2* methylation levels of 4 CpG sites by bisulfite sequencing 11β-HSD2 mRNA levels by qPCR	- Higher *HSD11B2* methylation levels at all 4 CpG in IUGR neonates - Inverse correlation between *HSD11B2* methylation at two CpG sites and 11β-HSD2 mRNA levels	Zhao et al. (2014) (282)
Total *n* = 131 - Preterm *n* = 54 ■SGA: 22.2% ■Male samples: 57.4% ■ACS: 40.7% ■Avg GA: 29.2 weeks - Term *n* = 77 ■SGA: 13% ■Male samples: 48% Avg GA: 38.8 weeks■	Whole blood -from UC vessels, or from a neonate’s vein/artery at NICU day 0	- GRα and GRβ mRNA isoforms by qPCR	- Higher GRβ and lower GRα/GRβ in SGA-preterm compared to AGA-preterm neonates - No effect of birthweight in term neonates	Go et al. (2013) (239)
Total *n* = 75 - VLBW preterm *n* = 43 ■Male samples: 53.5% ■SGA: 40% ■ACS: 86% Avg GA: 27.6 weeks■	CBMCs (day 0) and PBMCs at days 4–7 (only from preterm neonates)	- GRα and GRβ mRNA isoforms by RT-qPCR	- No effect of fetal sex, birthweight, ACS, or mode of delivery on GR mRNA levels	Yamamoto et al. (2022) (240)
Total *n* = 53 - Preterm ■SGA: 21% ■Male samples: 54.7% ■ACS: 100% GA range: 24–36 weeks■	UC blood + placenta	- Cortisol EIA and placental cytokine and *p*-glycoprotein (ABCB1) mRNA and protein expression by RT-qPCR and IHC	- Lower *p*-glycoprotein placental expression in SGA	Hodyl et al. (2013) (283)
Total *n* = 135 - All term - Healthy *n* = 53 ■SGA: ~ 10% - Asthmatic *n* = 82 ■SGA: 12.5% Avg GA: 39.5 weeks■	Placenta	- GR protein isoform levels by Western blot, cortisol levels by ELISA	- Increased GRβ and GRα-D1 protein in placentas of term SGA compared to term AGA	Saif et al. (2014) (244)
Total *n* = 110 - Preterm *n* = 55 ■ACS: 47.3% ■Male samples: 54.5% Avg GA: 33.3 weeks■	Placenta	- GR protein isoform levels by Western blot	- Lower GR A, GRα-D1, and GRα-D2 in SGA compared to AGA in female preterm samples, no effect in male preterm samples	Saif et al. (2015) (241)
Total *n* = 63 - IUGR preterm *n* = 23 ■Male samples: 43.5% ■GA range: 26–36 weeks - Healthy term AGA *n* = 40 ■Male samples: 50% GA range: 36–42 weeks■	Placenta	- Intranuclear GR protein levels, by IHC using antibodies against GRα and GRβ	- Higher GRα in female IUGR than in other groups - Higher GRβ protein in male IUGR than in other groups	Hutter et al. (2019) (284)
Intrauterine infection				
Total *n* = 33 - Preterm *n* = 22 Infection *n* = 11, ■Avg GA = 27.3 weeks ■No infection *n* = 11 ■Avg GA = 26.9 weeks - Term controls *n* =11 Avg GA = 37.0 weeks■	Amniotic fluid (amniocentesis at enrollment)	- Cortisol and DHEA by RIA	- Higher amniotic cortisol and DHEA levels in the infection group than no infection groups (preterm and term); *p* < 0.05	Gravett et al. (2000) (297)
Total *n* = 31 - Preterm neonates ■Infection *n* = 13 ■No infection *n* = 18 - GA range: 24–32 weeks - ACS: 93% neonates	Cord blood for ELISA Placental and amnion tissues to confirm infection	- Cortisol, IL-6, and IL-8 levels measured by ELISA	- Higher cord blood cortisol in infection group; *p* < 0.05 - Higher cord blood cortisol concentration in histological CA or funisitis compared to PCR bacterial detection alone - Positive correlation of cord blood cortisol with IL-6 (*r* = 0.64, *p* < 0.01) and IL-8 (*r* = 0.52, *p* < 0.01)	Miralles et al. (2008) (298)
Total *n* = 36 - All preterm neonates 4 groups: ■CA/ACS *n* = 6 ■CA/no-ACS *n* = 6 ■No-CA/ACS *n* = 12 ■No-CA/no-ACS *n* = 12 - GA range: 24–36 weeks	Placenta	- 11β-HSD1, 11β-HSD2, total GR, and GRα protein levels by Western blot	- Higher placental 11β-HSD1 protein expression in CA/no-ACS group than CA/ACS and no infection groups - Lower placental 11β-HSD2 protein expression in both CA groups compared to the no infection groups - No significant differences in placental GRα or total GR protein between groups	Johnstone et al. (2005) (299)
Total *n* = 16 - Term labor without infection *n* = 4 ■Avg GA 39.2 weeks - Preterm labor with infection/CA *n* = 8 ■Gram-negative pathogens *n* = 4 ■Other pathogens *n* = 4 ■Avg GA = 33.8 weeks	Amniotic tissue (membrane)	-11β-HSD1 protein levels by Western blot - *In vitro* stimulation of primary amniotic mesenchymal cells with LPS (10 ng/mL) and cortisol (1 µM) or both to the assay 11β-HSD1 expression	- Higher amniotic 11β-HSD1 protein expression in infection groups - Higher amniotic membrane 11β-HSD1 expression in gram-negative infection compared to other pathogens - Synergistic *in vitro* effect of LPS and cortisol on 11β-HSD1 protein upregulation in amniotic mesenchymal cells	Ling et al. (2024) (300)
Neonatal sepsis				
Total *n* = 90 - Term and preterm ■Septic term *n* = 30 •Avg GA = 38.0 weeks ■Septic preterm *n* = 30 •Avg GA = 34.1 weeks ■Healthy controls *n* = 30 •Avg GA = 35.3 weeks	Peripheral blood (collected at postnatal days 8–9)	-Serum ACTH and cortisol were measured at baseline (9 am and 9 pm) and after ACTH stimulation ■Absolute AI = < 6 µg/dL basal cortisol Relative AI = 7–15 µg/dL basal cortisol or < 9 µg/dL ∆ cortisol■	- Lower basal cortisol and ACTH levels in septic than healthy term controls - Septic neonates with AI: ■78.3% of term neonates ■83.3% of septic preterm - Blunted response to ACTH stimulation in both term and preterm septic neonates	Elsheikh et al. (2024) (316)
Total *n* = 60 - Term and near-term: ■Septic with circulatory collapse *n* = 30 ■Septic without circulatory collapse n = 30 - Avg GA = 38 wks	Peripheral blood (samples taken on postnatal days 4–14)	- Basal serum cortisol and precursors measured by LC-MS	- Similar serum cortisol levels in both groups, average lower than the literature-based reported range - Higher serum cortisol precursors (17-hydroxy-pregnenolone, pregnenolone, and progesterone) in neonates with circulatory collapse than without	Khashana et al. (2016) (317)
Total *n* = 30 -Term and preterm neonates - Average GA: 30 weeks - All patients with septic shock	Peripheral blood (samples taken at the onset of septic shock and after the ACTH test)	- Total basal and stimulated serum cortisol measured by electrochemiluminescence ■Absolute AI: baseline cortisol < 15 µg/dL and ∆ cortisol < 9 µg/dL Relative AI: baseline cortisol ≥ 15 µg/dL and ∆ cortisol < 9 µg/dL■	- 27% of septic neonates with AI: ■Absolute AI = 20% ■Relative AI = 6.67% - 57% septic neonates with low basal cortisol level but adequate ACTH response	Bhat et al. (2022) (318)
Total *n* = 35 - Term and late-preterm neonates with catecholamine-resistant septic shock	Peripheral blood (samples taken at the onset of septic shock)	- Total serum cortisol by unreported method ■ Absolute AI = < 6 µg/dL basal cortisol ■ Relative AI = 7–15 µg/dL basal cortisol	- 48.6% of catecholamine-resistant shock neonates with AI - Survival with hydrocortisone treatment in AI neonates - Higher mortality in serum cortisol levels (> 16 µg/dl) in septic associated with higher mortality than septic neonates with AI (*p* < 0.031)	Kumar et al. (2023) (319)
Total *n* = 60 - All preterm ■Sepsis with circulatory collapse *n* = 30 ■Sepsis without circulatory collapse *n* = 15 ■”Healthy” controls *n* = 15 ■GA range: 32–36 weeks	Peripheral blood (samples taken at 9 am and 9 pm, after NICU admission)	- Total serum cortisol and ACTH measured by electrochemiluminescence immunoassay ■Absolute AI = < 4 µg/dL basal cortisol or ∆ cortisol < 18 µg/mL	- Higher morning and evening cortisol levels in septic neonates (*p* < 0.03) - Significant HPA axis dysregulation in septic neonates with circulatory collapse ■50% with absent diurnal rhythm ■10% with reverse rhythm ■23% with relative AI ■16% with normal HPA axis function - Inverse correlation between cortisol levels and blood pressure in sepsis with circulatory collapse	Razik et al. (2021) (321)
Neonatal RDS/BPD				
Total *n* =11 - Preterm neonates ■RDS *n* = 6 ■No RDS *n* = 5 - GA range: 23–31 weeks	Lung, autopsied neonates 0–11 h after death	- GR number and affinity: radioactive DEX-binding assay	- DEX binding undetectable in tissues from RDS neonates - DEX binding detected in all non-RDS-related tissues	Ballard and Ballard (1974) (104)
Total *n* = 30 - Preterm neonates ■RDS *n* = 12 ■No-RDS *n* =18 - Avg GA: 32.42 weeks	PBMCs from preterm neonates (2–3 days old)	- GR number and affinity: radioactive DEX-binding assay	- Lower DEX binding in preterm neonates with RDS (*p* < 0.001)	Kerepesi and Arányi (1985) (237)
Total *n* = 20 - Preterm neonates ■RDS *n* = 7 ■No-RDS *n* = 13 - Avg GA: 30.62 weeks	CBMCs (at birth)	- GR number and affinity: radioactive DEX-binding assay	- No significant difference in DEX binding and affinity between RDS and non-RDS samples	Vlugt et al. (1997) (238)
Total *n* = 54 - Preterm neonates ■RDS *n* = 12 ■No-RDS *n* = 20 ■BPD *n* = 17 ■No-BPD *n* = 15 - Avg GA *n* = 29.2 weeks	Whole blood from UC, vein, or artery (at birth)	- GRα and GRβ mRNA isoforms by RT-qPCR	- No association of GRα, GRβ, and GRα/GRβ ratio at birth with RDS -4-fold lower GRα mRNA levels at birth in neonates later diagnosed with BPD compared to non-BPD (*p* = 0.069)	Go et al. (2013) (239)
Total *n* = 43 - VLBW preterm neonates ■RDS *n* = 32 ■No-RDS *n* = 11 - Avg GA: 27.6 weeks	CBMCs (at day 0) and PBMCs at days 4–7	- GRα and GRβ mRNA isoforms by RT-qPCR	- Similar GRα and GRβ mRNA levels and ratios at days 0 and 4–7 in RDS and no-RDS groups - Severity and onset of RDS at time of sampling not reported	Yamamoto et al. (2022) (240)
Total *n* = 41 - Preterm neonates ■RDS *n* = 18 ■No RDS *n* = 23 - Avg GA: 30 weeks	Umbilical cord blood CD14^+^ monocytes	- GRα and GRβ mRNA expression by RT-qPCR	- Lower GRα in neonates with RDS than those without RDS (*p* = 0.043) - No correlation between GRβ mRNA levels and RDS - Correlation between high GRβ expression and sepsis, transfusions, longer parenteral feeding, and longer hospital stay, all associated with	Ataseven et al. (2017) (326)

*AI*, adrenal insufficiency; *Avg*, average; *ACTH*, adrenocorticotropin hormone; *AGA*, average for gestational age; *CA*, chorioamnionitis; *CBMC*, cord blood mononuclear cells; *CRH*, corticotropin-releasing hormone; *DHEAS*, dehydroepiandrosterone sulfate; *FBG*, fasting blood glucose; *GA*, gestational age; *GR*, glucocorticoid receptor; *11β-HSD*, 11beta-hydroxysteroid dehydrogenase; *IUGR*, intrauterine growth restriction defined by umbilical cord artery doppler ultrasonography; *LC-MS*, liquid chromatography mass spectrometry; *LPS*, lipopolysaccharide; *PBMC*, peripheral blood mononuclear cells; *RDS*, respiratory distress syndrome; *RIA*, radioimmunoassay; *RT-PCR*, reverse transcription polymerase; *SGA*, small for gestational age defined as birthweight < 10th percentile; *UC*, umbilical cord; *VLBW*, very low birth weight defined as < 1,500 g.

#### 5.3.1 IUGR

Fetal glucocorticoid homeostasis dysregulation in IUGR is caused by the intrauterine stress of nutrient and oxygen insufficiency (267, 268). While supraphysiological cortisol levels alone can induce IUGR, as discussed in Section 4, increased fetal cortisol bioavailability is considered a consequence rather than a cause in the most common type of IUGR, which is characterized by late-onset placental insufficiency with brain-sparing effects (267, 268). However, interindividual variability in IUGR-associated glucocorticoid dysregulation is evident (**Table 3**). Importantly, most human studies define IUGR as birth weight < 10th percentile, also known as SGA. However, IUGR is better defined as the pathological counterpart of SGA, diagnosed by ultrasonographic assessment of estimated fetal weight < 10th percentile, in conjunction with abnormal umbilical artery Doppler velocimetry (268). Both IUGR and SGA have been associated with either higher or lower umbilical cord blood cortisol levels (145, 269–274). Interestingly, fetal cortisol levels in IUGR appear to be regulated in a gestational age-dependent manner. Preterm IUGR neonates exhibit higher cord blood cortisol levels than gestational age-matched AGA controls (269–271), while term IUGR neonates show the opposite effect (145, 273, 274). These discrepancies may be explained by gestational age-dependent differences in IUGR etiologies, placental development, and fetal HPA axis maturation (268, 275). Despite conflicting reports on cord blood cortisol levels, it is assumed that the IUGR fetus is exposed to supraphysiological cortisol levels, although the timing, degree, and duration of this exposure may vary (268, 275). Excess fetal glucocorticoid exposure, in turn, alters normal key organ developmental trajectories. Indeed, both preterm and term IUGR neonates have shown blunted HPA axis reactivity, demonstrating intrauterine stress-dependent glucocorticoid programming (276, 277). Furthermore, both preterm and term infants exhibit decreased activity and expression of the placental physiological barrier 11β-HSD2 (278–281), consistent with the proposed increased cortisol bioavailability. Interestingly, a stronger discrepancy in placental 11β-HSD2 expression between IUGR and AGA controls was observed in term neonates compared to very preterm neonates (278, 280), highlighting the interaction of gestational age with the fetal response to placental insufficiency. Decreased placental 11β-HSD2 expression and activity were not due to mutations or imprinting (281) but are proposed to be mediated by promoter hypermethylation (282). Much less is known about IUGR-mediated dysregulation of fetal tissue GR physiology. Preterm, but not term, SGA newborns exhibited increased GRβ mRNA levels in cord blood (239), although this finding was not confirmed in a separate study using CBMCs (240). However, another study found that term SGA newborns had higher placental GRβ protein levels than gestational age-matched controls (244). Furthermore, preterm SGA placentas displayed lower P-glycoprotein expression compared to gestational age-matched samples, suggesting higher glucocorticoid bioavailability in a fetal sex-independent manner (283). Finally, two studies demonstrated sexual dimorphism in IUGR-specific GR expression regulation. Saif et al. found decreased placental expression of the less sensitive GR isoforms GRα-D1 and GRα-D2 in preterm female SGA compared to female AGA, with no differences observed in preterm male samples (241). Hutter et al. found increased placental GRα protein levels in female samples and GRβ protein levels in male samples within the preterm IUGR cohort (284). These studies suggest that IUGR female samples exhibit higher glucocorticoid sensitivity due to expression of more sensitive GR protein isoforms.

Animal models of IUGR have confirmed dysregulation of glucocorticoid homeostasis due to placental insufficiency in a tissue- and time-dependent manner. Indeed, *in vivo* research has demonstrated downregulation of fetal brain drug transporters in term IUGR, suggesting increased brain-specific bioavailability that may explain the adverse neurodevelopmental outcomes observed in IUGR subjects exposed to ACS (285). Furthermore, worse pulmonary outcomes have been observed in IUGR models, although these findings depend on the type of stressor and timing of treatments relative to birth (285). For instance, uteroplacental embolization between 109 and 130 days, but not between 120 and 140 days, increased surfactant protein levels in near-term lambs (286, 287), although both IUGR models showed fetal pulmonary structural immaturity despite elevated fetal cortisol levels. In addition, a different IUGR model of placental restriction, induced by pre-pregnancy carunclectomy, showed an inverse relationship between fetal hypercortisolemia and pulmonary surfactant protein expression at late-preterm gestational ages (288). Finally, in a very preterm IUGR model, single uterine artery ligation resulted in increased fetal cortisol levels without maturational pulmonary effects (289). However, in this same model, ACS exposure unexpectedly increased fetal surfactant protein expression and decreased pulmonary cell proliferation without affecting pulmonary septation in both control and IUGR animals, suggesting a partial beneficial effect of ACS (289). Remarkably, ACS further inhibited growth in this model, suggesting an additive effect of betamethasone and cortisol. Overall, *in vivo* research clearly demonstrates that placental insufficiency leads to fetal cortisol overexposure, resulting in growth restriction without accompanying pulmonary maturation, indicating tissue-specific cortisol resistance in the lung.

#### 5.3.2 Maternal diabetes

Significant glucocorticoid homeostasis dysregulation has been observed in adult diabetic populations. For instance, both types I and II diabetes mellitus (T1/T2DM) have been associated with elevated cortisol levels and HPA axis hyperactivity (290, 291). Similarly, higher maternal cortisol levels have been reported in gestational diabetes patients compared to healthy controls (292, 293). This diabetes-induced subclinical hypercortisolism is thought to result from decreased hippocampal regulation of the HPA axis due to hyperglycemia-induced atrophy within this region (290). In addition, adult diabetic cohort studies have shown a reduced PBMC GRα/GRβ mRNA ratio and decreased phosphorylation of GR at S211, suggesting impaired immune cell–glucocorticoid sensitivity (294). Currently, the impact of maternal diabetes on human fetal/neonatal glucocorticoid sensitivity remains unknown. However, *in vivo* and *ex vivo* research suggest that maternal diabetes may potentially dysregulate fetal glucocorticoid sensitivity. For example, a sheep model of gestational diabetes demonstrated reduced surfactant protein production and expression of GR/11β-HSD1 in the fetal lung (295). Furthermore, insulin abolished glucocorticoid stimulation of surfactant production in co-cultures of rat lung fibroblasts and epithelial cells (296). These studies suggest an important role of maternal diabetes in reducing fetal/neonatal glucocorticoid sensitivity.

#### 5.3.3 Intrauterine infection

Increased cord blood and amniotic fluid cortisol levels have been reported in both preterm and term pregnancies complicated by chorioamnionitis (297, 298). Decreased 11β-HSD2 and increased 11β-HSD1 expression were found in placental and amniotic tissue of human pregnancies with intrauterine infection (299, 300). Additionally, *ex vivo* exposure of amniotic fibroblasts to cortisol, endotoxin, or both led to increased 11β-HSD1 expression (300). These findings suggest that intrauterine infection augments fetal cortisol bioavailability, potentially accelerating pulmonary maturation. Indeed, several clinical studies have reported a reduced risk of RDS in preterm newborns exposed to intrauterine infection (301–303). However, other studies have found no benefit—or even an increased risk—of RDS in neonates exposed to chorioamnionitis (304, 305). These conflicting results may be explained by the severity of infection: low-grade infection may prime the fetal lung for glucocorticoid action, while high-grade infection may antagonize glucocorticoid effects via increased systemic and pulmonary inflammation (306). Animal models have confirmed a causal relationship between intrauterine infection and fetal lung maturation (307–309). Interestingly, in sheep, endotoxin-induced lung maturational effects occurred without concomitant increases in fetal cortisol levels, suggesting alternative cortisol-independent mechanisms yet to be identified (307). Furthermore, endotoxin and betamethasone exhibited additive effects on lung maturation, with transcriptomic analysis revealing distinct gene regulation patterns between the two (308). While both treatments promoted mesenchymal thinning via cell death, endotoxin uniquely suppressed the expression of elastic fibers and induced chronic inflammatory stress, possibly disrupting lung maturation in the long term (308). These observations partly explain the paradox wherein chorioamnionitis reduces RDS risk but increases the risk of BPD (310, 311). Finally, the impact of chorioamnionitis on human perinatal tissue glucocorticoid sensitivity and GR expression remains an area of limited exploration. However, one report on preterm chorioamnionitis showed no difference in total GR or GRα protein levels, independent of ACS exposure (299).

#### 5.3.4 Neonatal sepsis

Similar to adult and pediatric sepsis, neonatal sepsis has been associated with glucocorticoid homeostasis dysregulation, manifested as either hypo- or hypercortisolism. Longitudinal studies in adults with sepsis have shown that glucocorticoid dysregulation begins with an initial rise in free cortisol, decreased hepatic CBG production, and ACTH–cortisol dissociation during the acute phase (312, 313). This is followed by adrenal insufficiency, characterized by decreased cortisol levels, suppression of the HPA axis, and reduced tissue glucocorticoid sensitivity in the subacute/chronic phase (312, 313). Notably, worse outcomes have been observed in sepsis patients with the highest initial elevation in free cortisol (314, 315). Findings on cortisol regulation in neonatal sepsis, however, remain inconclusive. While three independent studies reported very low cortisol levels (< 15 µg/dL) in most septic neonates (316–318), Kumar et al. reported significant variability, with ~ 50% of neonates presenting with low cortisol levels and the remainder showing elevated levels (319). Similar to adults, septic neonates with higher cortisol levels exhibited increased mortality (319). The association of hypercortisolemia with mortality in critically ill neonates was confirmed in a separate, larger study (320). Furthermore, another study found increased basal cortisol levels in the preterm septic neonates compared to controls, together with significant HPA axis dysfunction and an inverse relationship between blood pressure and cortisol levels, suggesting cardiovascular tissue glucocorticoid resistance (321). Moreover, a blunted cortisol synthesis pathway and impaired HPA axis response to ACTH have been observed in both preterm and term neonates with sepsis (316–318). It is important to note that preterm response to sepsis is further complicated by HPA axis immaturity. Therefore, while the onset of sepsis may induce an elevation of cortisol levels in adults, this response might be limited in neonates due to developmental constraints. Although tissue-specific GR physiology has not been studied in human neonatal sepsis, research in adults has shown reduced glucocorticoid sensitivity due to decreased GRα and increased GRβ expression in peripheral blood and liver (322). Moreover, decreased PBMC GR expression correlated with mortality in adult and pediatric sepsis (323, 324), highlighting the critical role of tissue glucocorticoid homeostasis in sepsis outcomes. Indeed, murine sepsis models further demonstrate that GR deletions in hepatic or endothelial cell lineages exacerbate inflammation and reduce survival (322, 325), underscoring the importance of tissue GR function in sepsis.

#### 5.3.5 Neonatal RDS

Reduced fetal/neonatal GR levels have been observed in neonatal pulmonary complications, particularly in RDS. In this context, Ballard and Ballard reported undetectable GR levels in autopsied lung samples from neonates with RDS (104). Later, Kerepesi and Arányi confirmed these findings, showing decreased dexamethasone-binding levels in preterm newborns with RDS compared to those with other morbidities, such as bronchopneumonia. Notably, they used PBMCs as a surrogate tissue for the lung (237), where GR levels correlated with RDS severity. In contrast, studies using CBMCs, collected prior to RDS development, did not reveal differences in GR number estimated by ligand-binding assays (238) or real-time PCR (239, 240) between RDS neonates and gestational age-matched controls. Only one study found a significant association between reduced GRα mRNA levels in umbilical cord CD14^+^ monocytes and later development of RDS (326), suggesting that this immune cell type is more sensitive to glucocorticoid dysregulation before RDS onset. These findings suggest that GR expression downregulation does not precede but rather occurs as a result of severe RDS, since decreased GR levels were not evident at birth. Similarly, reduced pulmonary and immune cell GR levels have been observed in critically ill pediatric and adult populations (327), although the mechanisms responsible for GR downregulation remain unknown.

## 6 Conclusions

It is well accepted that preterm fetuses and neonates are particularly vulnerable to intrauterine stressors, leading to dysregulation of cortisol metabolism, the HPA axis, and tissue GR. However, uncovering the specific stressors and determinants of glucocorticoid sensitivity that impact neonatal health remains challenging, likely due to confounding factors that hinder the interpretation of observational studies. Nevertheless, this review highlights research that, either deliberately or inadvertently, has revealed key determinants of perinatal glucocorticoid sensitivity. First, gene polymorphisms in drug-metabolizing enzymes and transporters have been associated with neonatal disease, suggesting that genetic factors influence synthetic glucocorticoid bioavailability. Second, lung tissue and immune cells were shown to express functional GRs that are unaffected by gestational age, fetal sex, or ACS exposure, suggesting that GR deficiency may not be a major determinant of glucocorticoid sensitivity in preterm fetuses. In contrast, the placenta has emerged as a more sensitive tissue to glucocorticoid dysregulation, influenced by gestational age, fetal sex, maternal BMI, and disease. This sensitivity may be explained by the essential role of the placenta in coordinating endocrine, growth, and metabolic functions in response to intrauterine environmental cues. Future research is needed to unveil the mechanisms and determinants of perinatal glucocorticoid sensitivity in additional, unexplored tissues such as umbilical artery endothelial, smooth muscle, and mesenchymal cells. Third, perinatal complications such as IUGR, chorioamnionitis, neonatal sepsis, and RDS have been associated with significant dysregulation of fetal/neonatal glucocorticoid homeostasis. Specifically, IUGR was associated with blunted HPA axis reactivity and increased cortisol bioavailability in preterm newborns, with further sexual dimorphism observed in placental GR isoform expression, where females exhibited increased expression of sensitive GR isoforms. Importantly, *in vivo* models of IUGR and gestational diabetes demonstrated reduced cortisol-mediated effects in the fetal lung, suggesting decreased pulmonary sensitivity to cortisol. Similar to IUGR, neonatal sepsis was also associated with blunted HPA axis reactivity in preterm newborns. Furthermore, higher cortisol levels in preterm newborns with sepsis are associated with circulatory collapse and death, suggesting decreased vascular sensitivity to cortisol. Severe neonatal RDS is associated with reduced pulmonary and CBMC expression of GR, indicating significant dysregulation of this receptor during the progression of the disease. In contrast, and uniquely, chorioamnionitis was significantly associated with increased glucocorticoid sensitivity in both animals and humans, although this effect may depend on the severity of the disease. Altogether, preterm birth and associated intrauterine stressors significantly impact perinatal glucocorticoid homeostasis, which can influence clinical outcomes and responses to both endogenous and synthetic glucocorticoids. Finally, we emphasize a critical knowledge gap regarding the role of the tissue environment as a modifier of glucocorticoid sensitivity and stress the need for integrated omics approaches to uncover tissue-specific epigenetic modifications associated with perinatal disease and clinical response to glucocorticoids. Such studies could advance our understanding of the molecular mechanisms underlying GR homeostasis dysregulation in various pregnancy disorders and lead to novel strategies to prevent or reverse these molecular changes, ultimately paving the way for optimized perinatal glucocorticoid therapies in the era of precision medicine.
